# Development and validation of a machine learning clinicogenomic model to improve prognostic stratification in ER-positive/HER2-negative early breast cancer

**DOI:** 10.1186/s13058-026-02388-4

**Published:** 2026-09-16

**Authors:** Michail Sarafidis, Emmanouil G. Sifakis, Jonas Bergh, Theodoros Foukakis, Alexios Matikas

**Affiliations:** 1 Department of Oncology-Pathology, Karolinska Institutet, Visionsgatan 4, 171 64 Stockholm, Sweden; 2 Breast Center, Theme Cancer, Karolinska Comprehensive Cancer Center, Karolinska University Hospital, Stockholm, Sweden

**Keywords:** ER-positive/HER2-negative breast cancer, Early breast cancer, Prognostic stratification, Risk prediction, Machine learning, Random survival forest, Clinicogenomic model, Gene expression signatures, 21-gene recurrence score

## Abstract

**Background:**

Several gene expression signatures (GESs) are used for risk stratification in estrogen receptor-positive/human epidermal growth factor receptor 2-negative (ER-positive/HER2-negative) early breast cancer. Recent integrative approaches combine tumour proliferation, estrogen receptor signalling, immune activity, and clinicopathologic features, yielding gains in prognostic accuracy. We therefore developed a machine-learning-based clinicogenomic prognostic model integrating a research-grade implementation of an established GES with immune-related and clinicopathologic features to improve prognostic stratification in ER-positive/HER2-negative early breast cancer.

**Methods:**

This retrospective integrative analysis of publicly available and institutional microarray and RNA-sequencing data included four independent datasets of systemically untreated or endocrine-treated patients (n = 5132). One dataset was used for the development of a random survival forest (RSF) model, and three datasets were used for external validation. The RSF model incorporated a research-grade implementation of the 21-gene recurrence score (RS), the 14-gene immunoglobulin signature, and clinicopathologic features. Model performance was evaluated relative to the standalone research-grade implementation of the 21-gene RS using measures of risk stratification, discrimination, and prediction error. Feature contributions were examined using time-dependent explainability analyses.

**Results:**

In external validation, the RSF model identified larger low-risk groups than the standalone 21-gene RS, with relative increases of 33.8 to 111.8%, while maintaining similar or higher horizon-specific survival estimates. The RSF model also improved discrimination over the standalone 21-gene RS across validation datasets, with absolute increases of 0.035 to 0.058 in the concordance index and 0.030 to 0.064 in the integrated area under the time-dependent receiver operating characteristic curve. Risk separation, measured by the difference in restricted mean survival time, was consistently greater for the RSF model, whereas prediction error was comparable or lower according to Cox-recalibrated integrated Brier score. Explainability analyses indicated time-dependent feature contributions, with the 21-gene RS and clinicopathologic variables driving early prognostic performance and the immune component contributing a smaller but more stable effect over time.

**Conclusions:**

This explainable clinicogenomic machine learning model integrates research-grade molecular, immune-related, and clinicopathologic features and improves prognostic stratification in retrospective ER-positive/HER2-negative early breast cancer datasets. These findings support its potential as a more informative and biologically grounded approach to early breast cancer risk assessment.

**Supplementary Information:**

The online version contains supplementary material available at https://doi.org/10.1186/s13058-026-02388-4.

## Background

Gene expression signatures (GESs) are recommended to help guide decisions on adjuvant chemotherapy for primary resected estrogen receptor-positive (ER-positive) and human epidermal growth factor receptor 2 negative (HER2-negative) early breast cancer [1, 2]. These tools are strongly prognostic, since they identify patients at such low risk for recurrence, that adjuvant chemotherapy is not expected to provide clinically meaningful benefit [3]. While their predictive value has been demonstrated in retrospective analyses of prospective studies [4–7], the only prospective randomised trial specifically designed to test the interaction between the 21-gene recurrence score (RS) and chemotherapy did not confirm a predictive effect [8]. As such, the clinical utility of GESs largely depends on their prognostic value.

The molecular drivers captured by widely used GESs differ across available assays, but generally fall into two main biological processes, proliferation and estrogen receptor signalling [9, 10]. Although head-to-head comparisons are limited, commercially available GESs that also integrate clinicopathologic information, such as *Prosigna* (Veracyte, South San Francisco, CA, USA), *RSClin* (Exact Sciences, Madison, WI, USA) and *EPClin* (Myriad Genetics, Salt Lake City, UT, USA), seem to outperform tools based solely on gene expression [11–13]. In addition, we have previously shown that immune gene expression is prognostic in ER-positive/HER2-negative breast cancer [14, 15], and provides additional prognostic information to commercially available GESs [16]. As such, novel tools that integrate information from all those sources, i.e. proliferation, endocrine sensitivity, immune function, and clinicopathologic variables have been developed and successfully deployed [17, 18], demonstrating the feasibility and clinical validity of more complex profiling tools.

However, this increasing complexity raises a critical unanswered question: how best to integrate features from different data sources into a single interpretable and explainable model suitable for routine clinical use. Survival-oriented machine learning approaches provide an effective tool for leveraging heterogeneous biological and clinicopathologic information in a unified manner [19]. Recent studies have further highlighted the potential of multimodal machine learning frameworks to holistically integrate clinicogenomic data for patient comparison and disease prognosis [20]. Thus, there is growing evidence that multimodal models may support clinicians with more accurate and individualised risk stratification while preserving clinical interpretability and prognostic relevance.

Here, we developed and externally validated a prognostic clinicogenomic machine learning model for early-stage ER-positive/HER2-negative breast cancer, evaluating whether it improves prognostic performance relative to the standalone research-grade implementation of the 21-gene RS while providing explainable feature-level insights into the biological and clinical determinants of risk.

## Methods

### Study design

This retrospective cohort study was designed to develop and externally validate an integrative clinicogenomic machine learning model for patients with early-stage ER-positive/HER2-negative breast cancer who had not received (neo)adjuvant chemotherapy using transcriptomic and clinicopathologic data. All analyses adhered to the STROBE and REMARK guidelines for observational and biomarker studies, respectively [21, 22], and completed checklists are provided in the Supplementary Material. The Ethics Committee in Stockholm approved the gene expression analyses conducted in the three cohorts comprising the institutional Stockholm Breast Cancer Cohorts dataset (dnr 2006/394-31/3, 2006/1183-31/2 and amendments 2018/789-32, 2018/790-32; dnr 148-93 and amendment 2002/02-260; dnr 2001/01-139). All other gene expression and clinical data were obtained from publicly available repositories, and no additional ethics permits were required.

**Fig. 1:**
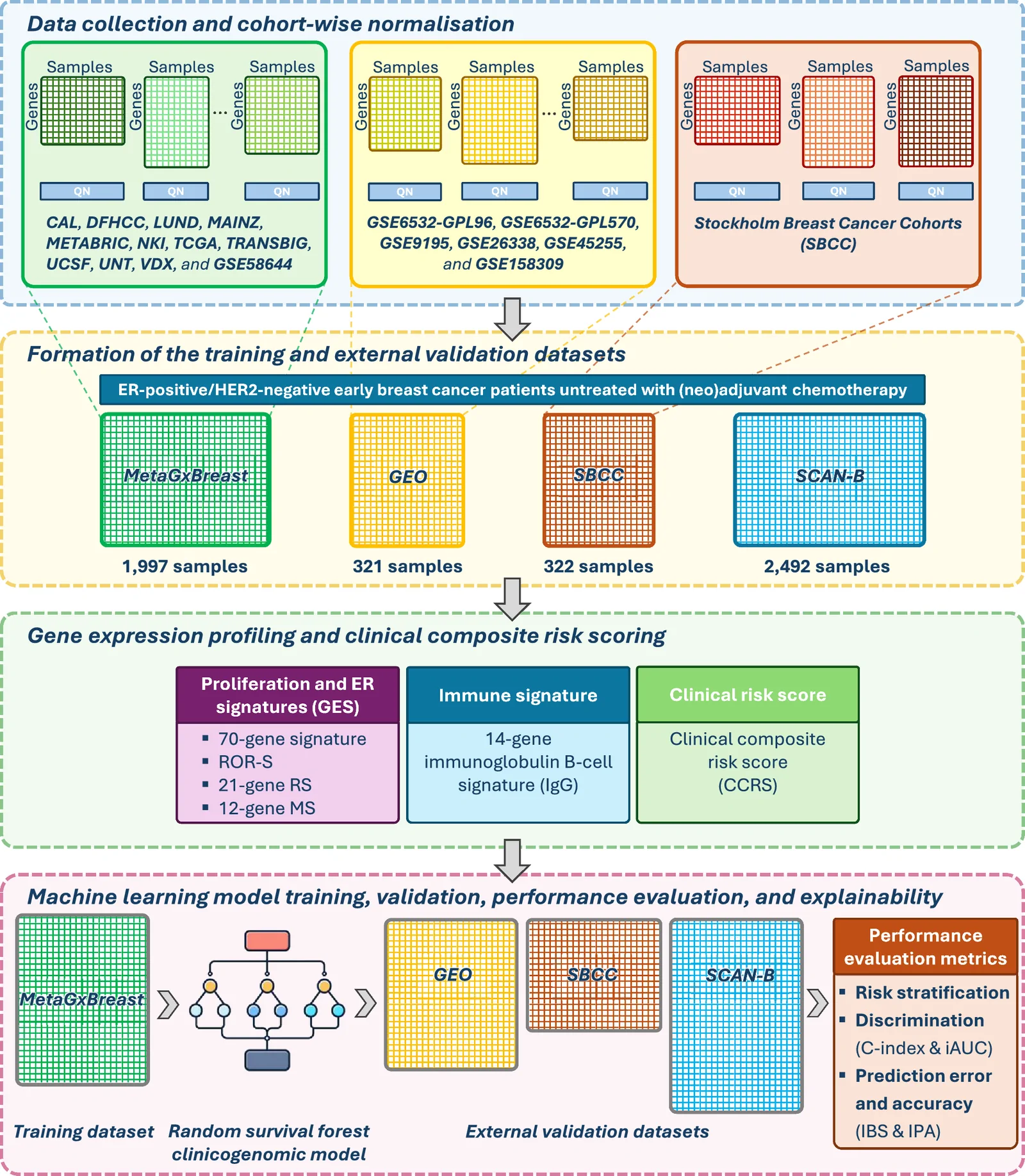
Overall study design. The study comprised four main steps: (1) data collection, cohort-wise preprocessing, and normalisation; (2) construction of the *MetaGxBreast* training dataset and external validation datasets; (3) calculation of gene expression signatures and clinical composite risk score; and (4) Random survival forest (RSF) model development, external validation, performance evaluation, and explainability analysis

Figure 1 shows the overall design of this study, which included data collection, preprocessing and normalisation, dataset formation, gene expression profiling, clinical risk scoring, feature selection, model development, and validation.

### Study cohorts and sample selection

The training data for this study were derived from the R package *MetaGxBreast* [23], which encompasses curated and processed transcriptomic and clinical data from 39 breast cancer cohorts, comprising a total of 9717 tumour and normal tissue samples. In addition to the *MetaGxBreast* training compendium, multiple external cohorts were incorporated for independent validation. These were consolidated into three datasets: (i) the Gene Expression Omnibus (*GEO*) dataset, comprising six GEO-derived cohorts which were curated, harmonised, and merged into a unified dataset, (ii) the Stockholm Breast Cancer Cohorts (*SBCC*) dataset, combining a high-risk cohort for distant metastasis [24] and two population-based cohorts [25], which were processed similarly to *GEO*, and (iii) the Swedish Cancerome Analysis Network – Breast (*SCAN-B*) dataset, consisting of the corresponding population-based cohort [26]. The *GEO* dataset comprised the following accessions: *GSE6532-GPL96*, *GSE6532-GPL570*, *GSE9195*, *GSE26338*, *GSE45255*, and *GSE158309*.

To minimise sample redundancy and data leakage, all cohorts were systematically reviewed before inclusion using original study publications, institutional origin, recruitment period, cohort description, GEO metadata, and available sample identifiers. Particular attention was given to cohorts originating from the same institutions or overlapping study populations. When potential overlap could not be excluded, only one representation of the corresponding samples or cohort source was retained for analysis. In addition, several cohorts required clinical data enrichment or harmonisation from original publications and public repositories to recover missing clinicopathologic or follow-up data. Detailed information on each cohort’s institutional origin, sample numbers, sampling years, clinical data enrichment, and harmonisation procedures is provided in the Supplementary Methods and Supplementary Table 1.

Within each cohort, tumour samples were restricted to ER-positive/HER2-negative cases from patients who had received no systemic therapy or adjuvant endocrine therapy alone; patients who had received (neo)adjuvant chemotherapy or any other systemic therapy were excluded. Samples were required to have available follow-up data for distant metastasis-free survival (DMFS) or a related recurrence endpoint, and complete data for age, tumour size, histological grade, and nodal status. ER status was primarily defined by immunohistochemistry (IHC) when available. The only exception was the *GSE158309* dataset, for which ER status was inferred from *ESR1* expression, in accordance with the original publication [27], using the *bimod* function in the *genefu* R package [28], which models a bimodal expression distribution by fitting a mixture of two Gaussians. For HER2 status, clinical HER2 annotation was prioritised when available, whereas missing clinical HER2 status was inferred from the *ERBB2* expression distribution within each cohort using the *bimod* function. Expression-based HER2 inference was performed only in original cohorts that included at least 20 tumour samples and at least one identifiable *ERBB2* probe. For *MAINZ* and TRANSBIG cohorts, cohort-specific *ERBB2* probes were used following quality-control review of the automatic probe selection.

### Expression harmonisation and cohort-wise normalisation

Expression matrices were processed separately for each original cohort before gene signature scoring. For each cohort, expression data were restricted to the final model-eligible samples. Probe-level annotations were mapped to Entrez Gene IDs using curated platform-specific annotations, with updated mappings obtained from *org.Hs.eg.db* and/or *biomaRt* when required [29]. When multiple probes mapped to the same Entrez Gene ID, probes were collapsed by selecting a single representative probe per gene, prioritising the probe with the highest interquartile range (IQR), followed by fewer missing values and higher variance in the event of ties. This produced one gene-level expression matrix per original cohort, with unique Entrez Gene IDs as rows and eligible samples as columns.

Primary expression preprocessing was then performed independently within each cohort matrix. Genes were retained only if expression values were available in at least 50% of samples and in at least five samples within that cohort. Remaining missing values among retained genes were imputed using the gene-specific median calculated within the same cohort. Microarray expression matrices were then quantile-normalised (QN) within each cohort. For SCAN-B, the supplied Fragments Per Kilobase per Million reads (FPKM) values were transformed as log_2_(FPKM + 1), and no additional quantile normalisation was applied. The resulting platform-specific expression matrices were used for downstream gene signature scoring, model development, validation, and performance analyses.

### Gene expression signatures and clinical composite risk scoring

We calculated research-grade implementations of several widely used GESs using established R packages. The risk of relapse (ROR) score based on the intrinsic subtypes (ROR-S) was derived using the *BreastSubtypeR* package [30]. The 21-gene RS [31], the Amsterdam 70-gene signature [32], and the 12-gene molecular score (MS) [33] were calculated using the *genefu* R package [28]. These signatures were computed from the platform-specific preprocessed expression matrices. For the 21-gene RS and 12-gene MS, reference genes were not included in the computation. When one or two non-reference genes were unavailable after preprocessing, the corresponding score was recalculated using the remaining genes; scores were considered non-evaluable when more than two score genes were missing. The 14-gene immunoglobulin B-cell (IgG) signature [34] was computed as the mean expression of its available constituent genes using the gene-wise standardised version of each platform-specific preprocessed expression matrix. For all signatures, only genes retained after preprocessing and present within each cohort were used. Low- and high-risk groups for each GES were defined according to the default stratification provided by the respective implementation. For the implementations returning three risk categories, namely the 21-gene RS and ROR-S, the intermediate and high categories were combined into a single high-risk group.

To incorporate clinicopathologic information into the prognostic model, a clinical composite risk score (CCRS) was constructed following the principles described by Gelber et al. [35]. The CCRS included four clinicopathologic characteristics: age, tumour size, histological grade, and nodal status. The same reference subgroups as in the original publication were applied across all cohorts, while histological grade used grade I as the reference category. Univariable Cox proportional hazards models were fitted in the *MetaGxBreast* training dataset for each clinicopathologic characteristic, and the resulting training-derived log-hazard ratios (LogHRs) and centring constant were applied unchanged to the three validation datasets. For each patient, the CCRS was calculated by assigning the corresponding subgroup-specific LogHR for each clinical characteristic, assigning 0 to the reference level, summing the four values, and centring the result by subtracting the mean CCRS of the training dataset. Thus, a CCRS of 0 represents average clinical risk in the training dataset; higher values indicate increased clinical risk, whereas negative values indicate lower-than-average clinical risk.

### Prognostic feature assessment and aggregate comparator

The four GESs computed in this study primarily capture tumour proliferation and estrogen receptor signalling. To evaluate whether incorporating biologically distinct information could improve prognostic performance, we examined the independent prognostic value of all candidate features (GESs, IgG signature, and CCRS).

In the *MetaGxBreast* training dataset, the prognostic value of each feature was initially assessed using Kaplan–Meier curves and log-rank tests after dichotomising each continuous feature at its median within the training dataset. Separate multivariable Cox models comprising each candidate GES with the other two features were then fitted to assess their independent prognostic associations. All three features in each model were standardised (mean-centred and scaled by their standard deviations) to facilitate comparison of hazard ratios (HRs) across features. Pairwise Pearson correlations were also examined to assess redundancy and inter-feature dependence.

To integrate complementary prognostic information, we developed a clinicogenomic modelling framework designed to combine an established GES with the IgG signature and the CCRS. In this study, the framework was implemented using the 21-gene RS as the selected GES component because it showed the most precise independent association with DMFS in the training dataset, as indicated by the narrowest 95% confidence interval (95% CI). Because the absolute range of the calculated 21-gene RS differed substantially across expression platforms and cohorts [36], the model used a cohort-percentile version of the 21-gene RS rather than the raw score. This transformation was applied to preserve the within-cohort prognostic ranking of the signature while reducing scale differences introduced by platform-specific expression distributions and preprocessing. The final RSF model therefore comprised three features: the cohort-percentile 21-gene RS, the IgG signature, and the CCRS.

Finally, we derived an average clinicogenomic risk (ACGR) score as a simple aggregate comparator using the same three model inputs. Before aggregation, the cohort-percentile 21-gene RS, IgG signature, and CCRS were standardised to Z-scores using the means and standard deviations estimated in the *MetaGxBreast* training dataset. These training-derived parameters were applied unchanged to all validation datasets. Because higher IgG values indicate better survival, the standardised IgG score was multiplied by − 1. ACGR was then calculated as the arithmetic mean of the three risk-oriented standardised components, such that higher values indicate worse prognosis.

### Model training

The final analysis population included only patients with available follow-up data for DMFS or the applicable recurrence endpoint, complete data for all four clinical variables required for the CCRS, and treatment information. Survival times were administratively censored at 15 years for the *MetaGxBreast*, *GEO*, and *SBCC* datasets, and at 10 years for the *SCAN-B* cohort due to the absence of follow-up data beyond that time.

To model DMFS, a random survival forest (RSF) model was developed using the *ranger* R package [37]. The RSF model was trained using three input features: the cohort-percentile 21-gene RS (GES), the IgG signature, and the CCRS. Before model fitting, these features were standardised to Z-scores using the means and standard deviations estimated in the *MetaGxBreast* training dataset. Following the data-driven selection of the 21-gene RS as the GES component, no further feature elimination was performed because all three model inputs showed independent prognostic value in the training dataset.

The RSF model used a bootstrap sampling fraction of 63.2% per tree and the “*extratrees*” splitting rule, which randomly samples candidate split points to increase model randomisation and reduce overfitting. Hyperparameters governing forest size and complexity were selected using out-of-bag (OOB) Harrell’s concordance index (C-index) in the training dataset. Forest size (*num.trees*) was evaluated over a grid of 500 to 2000 trees, and maximum tree depth (*max.depth*) over values of 5 to 15. The combination with the highest OOB C-index was selected, with ties resolved in favour of the simpler model. The number of variables considered at each split (*mtry*) was set to the rounded-down square root of the number of features, and the minimum terminal node size (*min.node.size*) was set to the larger of 20 observations or 2% of the training sample size. Unless otherwise specified, default *ranger* settings were used for all remaining parameters.

The trained RSF model was applied to the training and all external validation datasets to generate predicted survival probabilities and corresponding absolute risks at 10 or 15 years, without any retraining or modification of model parameters. To stratify patients into low- and high-risk groups based on the ACGR score and the model-predicted risk, score-specific cut-offs were defined as the lower tertile of the corresponding continuous score distribution in the *MetaGxBreast* training dataset. Patients with scores at or below the training-derived cut-off were classified as low risk, whereas all remaining patients were classified as high risk. The training-derived numerical cut-offs were then applied unchanged to validation datasets, without recalculating dataset-specific tertiles.

### Model validation and performance evaluation

After defining the stratification cut-offs, model performance was assessed using a comprehensive set of evaluation metrics that capture risk stratification, discrimination, prediction error, and descriptive horizon-specific calibration. The RSF model was compared with the corresponding GES used as model input and with the ACGR score. In addition, the risk-stratification performance of the RSF model was compared with the other GESs that were not included as input features for the model.

Risk stratification was assessed by the number and proportion of patients classified as low risk, together with the Kaplan–Meier survival estimate and 95% CI for the low-risk group at the dataset-specific evaluation horizon. Group separation was further summarised using Cox model hazard ratios, log-rank tests, and the restricted mean survival time difference (ΔRMST) between low- and high-risk groups, calculated as RMST_low_ − RMST_high_ [38]. Because ΔRMST depends on the selected time horizon, values from *SCAN-B*, evaluated at 10 years, were interpreted separately from the 15-year estimates in *MetaGxBreast*, *GEO*, and *SBCC*.

Discrimination, describing the ability to distinguish patients experiencing earlier events from those remaining event-free, was evaluated using the C-index and the integrated area under the time-dependent receiver operating characteristic curve (iAUC). For multi-cohort datasets, the C-index was calculated with cohort stratification to avoid non-comparable cross-cohort risk-set comparisons. Prediction error was assessed using an inverse probability of censoring-weighted integrated Brier score (IBS), after recalibrating each continuous score using a univariable Cox model fitted within the corresponding original cohort. Only original cohorts with at least 20 evaluable patients and at least five events contributed to the iAUC, IBS, and IPA calculations. The iAUC and IBS were calculated on a 0.5-year grid from 0.5 years to the earlier of the dataset-specific evaluation horizon and the last observed event time; iAUC was aggregated using event-count weighting, whereas IBS was aggregated using sample-size weighting. The index of prediction accuracy (IPA) was calculated within each cohort as 1 − IBS_score_/IBS_KM_, where IBS_KM_ was the IBS of the covariate-free Kaplan–Meier reference prediction; cohort-specific IPA values were then aggregated using sample-size weighting. Because Cox recalibration was fitted and evaluated within the same cohorts, the resulting IBS and IPA values were regarded as apparent score-level comparative metrics rather than measures of native RSF absolute-risk calibration. Native RSF calibration was assessed separately using horizon-specific calibration plots.

The 95% CIs for C-index, iAUC, IBS, KM-reference IBS, and IPA were estimated using 1000 bootstrap resamples within each original cohort, preserving the cohort structure of the multi-cohort datasets. Paired bootstrap comparisons were used to compare the RSF model with the GES-only and ACGR comparators; for C-index and iAUC, positive RSF-minus-comparator differences favoured the RSF model, whereas for IBS, positive comparator-minus-RSF differences favoured the RSF model because lower IBS indicates better prediction accuracy.

### Cohort heterogeneity and sensitivity analyses

Because the training dataset and two of the three validation datasets were assembled from multiple original cohorts, we performed additional sensitivity and benchmarking analyses to evaluate the influence of cohort heterogeneity on model performance and risk stratification.

First, within the *MetaGxBreast* training compendium, we assessed heterogeneity across the original constituent cohorts by summarising cohort-specific sample size, event rate, median and maximum follow-up, and Kaplan–Meier survival estimates at 5, 10, and 15 years. Cohort-specific Kaplan–Meier curves were generated to visualise differences in outcome distributions across the original *MetaGxBreast* cohorts, and a descriptive log-rank test was used to assess survival heterogeneity. To evaluate whether apparent discrimination in the training dataset was influenced by between-cohort outcome differences, RSF discrimination was estimated using both pooled, unstratified and cohort-stratified C-index estimates, and the difference between them was reported.

We next examined whether differences in the proportion of patients classified as low risk across the training and validation datasets reflected differences in dataset composition and baseline prognosis. For each dataset, we summarised key clinicopathologic characteristics, treatment status, follow-up duration, event rate, Kaplan–Meier survival at the evaluation horizon, and the distribution of model-predicted risk. These descriptive analyses were used to contextualise variation in RSF-defined low-risk proportions across datasets, particularly in *SCAN-B*, which had a lower-risk clinicopathologic profile and a shorter evaluation horizon than the other datasets. Given the small number of datasets (n = 4), associations between the RSF-defined low-risk proportion and Kaplan–Meier-estimated baseline survival at common 5- and 10-year horizons were examined descriptively using Spearman rank correlations, without formal inference.

Dedicated subgroup analyses were then performed within *SCAN-B*, as this was the largest validation dataset and showed the most distinct clinicopathologic composition. Patients were stratified by age (< 65 versus ≥ 65 years) and tumour size (< 2 versus ≥ 2 cm). Within each subgroup, the trained RSF model was applied without retraining, recalibration, or modification of model parameters, and the same training-derived cut-offs used in the primary analysis were applied unchanged. For each subgroup, we summarised the number and proportion of patients classified as low risk, the 10-year Kaplan–Meier survival estimate for the RSF-defined low-risk group, Cox-model hazard ratios for high versus low RSF risk, C-index, and horizon-specific 10-year time-dependent AUC. Interaction tests were performed using both the dichotomised RSF risk group and the continuous RSF-predicted risk to assess whether the association between RSF risk and outcome differed by age or tumour-size subgroup.

Because the selected study population included both systemically untreated patients and patients treated with endocrine therapy alone, we additionally performed a treatment-stratified sensitivity analysis in *SCAN-B*. Patients were analysed separately according to untreated versus endocrine-treated status. The trained RSF model, predicted risks, and training-derived cut-offs were kept unchanged. Because this analysis paralleled the primary *SCAN-B* validation framework, model performance within each treatment subgroup was summarised using the main validation metrics, including low-risk survival, C-index, iAUC, IBS, and ΔRMST. Interaction tests were also performed using both the dichotomised RSF risk group and the continuous RSF-predicted risk to assess whether the association between RSF risk and outcome differed by treatment subgroup.

### Benchmark comparison against an *RSClin*-analogous model

We further benchmarked the RSF model against an established clinicogenomic prognostic framework by constructing an *RSClin*-analogous Cox comparator among node-negative patients. This comparator was not intended to reproduce the commercial *RSClin* tool, but to provide a transparent benchmark using the published *RSClin* covariate structure: 21-gene RS, age, tumour size, and histological grade [12]. The 21-gene RS and age were modelled using two-degree-of-freedom natural cubic splines, tumour size was modelled as a continuous linear covariate truncated to 0.5–5 cm, and histological grade was modelled as a categorical covariate. Unlike the original tool, which derives risk through a patient-specific meta-analysis across trial cohorts with trial-based baseline hazards, our comparator was a single Cox model fitted in the node-negative *MetaGxBreast* training subset. It therefore reproduces the *RSClin* covariate model rather than the *RSClin* risk estimates themselves. It was then applied unchanged to node-negative subsets of *GEO*, *SBCC*, and *SCAN-B*. Because *RSClin* estimates 10-year distant recurrence risk, all head-to-head discrimination analyses involving the *RSClin*-analogous comparator were evaluated at a common 10-year horizon. For these analyses, follow-up in *MetaGxBreast*, *GEO*, and *SBCC* was administratively censored at 10 years, whereas *SCAN-B* was already evaluated at 10 years. Dedicated 10-year RSF risk predictions were generated for the same node-negative patients. The *RSClin*-analogous score was compared with the 21-gene RS and the RSF model using cohort-stratified C-index estimates within the node-negative subsets.

To evaluate whether the IgG signature added prognostic information beyond this clinicogenomic benchmark, a base *RSClin*-analogous model and an IgG-extended *RSClin*-analogous model were refitted on the same node-negative training subset. The nested models were compared using a likelihood-ratio test (LRT) and the corresponding change in C-index. Both fitted models were then applied unchanged to the corresponding node-negative, IgG-complete validation subsets, with their 10-year C-indices compared on identical patient sets. The nested Cox models and the LRT used the prespecified training follow-up, whereas all reported event counts and C-index estimates were evaluated at the common 10-year horizon.

### Model explainability

Because explainability is essential for transparent machine learning workflows, we complemented the performance evaluation with dedicated model-diagnostic and explainability analyses of the trained RSF model in the *MetaGxBreast* training dataset, using the *survex* R package [39]. Model-level diagnostics were used to assess global RSF behaviour over time, including time-resolved prediction error, cumulative/dynamic discrimination, and residual patterns. Variable importance was assessed using permutation-based feature-importance analyses to quantify the relative contribution of each model input to prognostic performance. Time-dependent feature contributions were further examined using SurvSHAP(t) summaries [40], while partial-dependence plots were used to visualise how changes in individual features influenced the model’s average predicted survival function over time. Together, these analyses provide complementary insight into overall model behaviour, the relative importance of each predictor, and the directionality and time-dependence of feature effects.

### Statistical methods

The final analysis population included 5132 patients: 1997 in *MetaGxBreast*, 321 in *GEO*, 322 in *SBCC*, and 2492 in *SCAN-B*. The selected evaluation horizon was 15 years for *MetaGxBreast*, *GEO*, and *SBCC*, and 10 years for *SCAN-B*. Events within the selected horizon were 446, 52, 96, and 108, respectively, and median follow-up was 9.6, 12.2, 15.0, and 5.3 years, respectively. The *MetaGxBreast* training dataset therefore provided a large number of observations and events for RSF model development, while the three validation datasets enabled external assessment across datasets with differing sample size, follow-up duration, and event burden. Dataset sizes, event counts, follow-up summaries, treatment status, and clinicopathologic distributions are provided in Table 1. Follow-up duration was summarised using the reverse Kaplan–Meier method. Differences in categorical clinicopathologic characteristics across datasets were assessed using Pearson’s chi-square test, with Fisher’s exact test used where appropriate for sparse tables. Bias-corrected Cramér’s V was calculated as an effect-size measure for cross-dataset differences in categorical variables. Continuous variables and model scores were summarised using medians and IQRs and compared between groups using the Wilcoxon rank-sum test where applicable. For the three RSF input features, *p* values from pairwise cross-dataset Wilcoxon rank-sum tests were adjusted using the Benjamini–Hochberg method. Pairwise associations among continuous scores were quantified using Pearson correlation coefficients.

The primary endpoint was DMFS, defined as the time from diagnosis to distant metastasis or death, whichever occurred first. When DMFS was not available, the closest available recurrence endpoint was used, as defined for each cohort. Specifically, two of the three *SBCC* cohorts were analysed using distant relapse-free survival (DRFS), relapse-free survival (RFS) was used for the *GEO* cohort *GSE26338*, and distant recurrence-free interval (DRFi) was used for *SCAN-B*. Patients without an event were censored at last follow-up or administratively censored at the selected analysis horizon.

Survival probabilities at fixed time points were estimated using the Kaplan–Meier method and reported with 95% CIs. Differences between survival curves were assessed using the log-rank test. Cox proportional hazards models were used to estimate associations with DMFS, reporting HRs with 95% CIs and Wald *p* values. For the multivariable feature-assessment Cox models, the proportional hazards assumption was assessed using Schoenfeld residuals with the *cox.zph* function, and the linearity of continuous covariates was examined using martingale residual plots.

Model performance was evaluated using complementary metrics for risk stratification, discrimination, prediction error, and calibration, as described in the “Model validation and performance evaluation” section, together with bootstrap-based confidence intervals and paired model comparisons.

Heterogeneity across the original *MetaGxBreast* cohorts was evaluated using cohort-specific survival summaries and log-rank testing, together with comparison of pooled unstratified and cohort-stratified C-index estimates. The association between dataset-level baseline DMFS and the RSF-defined low-risk fraction was examined descriptively using Spearman rank correlation, without formal inference because of the small number of datasets. The incremental prognostic value of additional terms, including subgroup interaction terms and IgG in the *RSClin*-analogous benchmark, was evaluated using an LRT between nested Cox models fitted on the same analysis subset, complemented by the corresponding change in C-index where applicable.

All statistical tests were two-sided, and *p* < 0.05 was considered statistically significant. No multiplicity correction was applied to secondary sensitivity, subgroup, or benchmark analyses; these analyses were interpreted descriptively. Fixed random seeds were used for reproducibility.

All analyses were conducted in R v4.6.0, using relevant Bioconductor and CRAN packages.

## Results

### Cohort characteristics and cohort-wise gene-expression profiling

A total of 5132 tumour samples from patients with early-stage ER-positive/HER2-negative breast cancer, untreated with (neo)adjuvant systemic therapy or only treated with adjuvant endocrine therapy were included in the final analysis. These data originated from four datasets: *MetaGxBreast* (n = 1997), *GEO* (n = 321), *SBCC* (n = 322), and *SCAN-B* (n = 2492). A detailed breakdown of included samples within each dataset is presented in the sample flow diagram (Supplementary Fig. 1), and analytical descriptions of individual cohorts are provided in Supplementary Table 1. All clinicopathologic characteristics for each final dataset, together with median follow-up times and crude DMFS event rates within the selected time horizons, are summarised in Table 1. Event rates varied modestly across the four datasets, reflecting expected differences in cohort composition and clinical context, and providing a heterogeneous setting appropriate for evaluating the generalisability of the prognostic model.

After cohort-wise annotation harmonisation and probe-to-gene collapsing, expression matrices were generated separately for the 12 original *MetaGxBreast* cohorts contributing to the final training dataset. Following cohort-specific gene-coverage filtering, the number of retained Entrez-annotated genes ranged from 6164 to 24,924 across cohorts, with a median of 13,266 genes. Remaining missing expression values among retained genes were imputed using the corresponding within-cohort gene median, after which appropriate normalisation was applied independently within each cohort. All selected gene-expression signatures, including ROR-S, the 21-gene RS, the Amsterdam 70-gene signature, the 12-gene MS, and the 14-gene IgG signature, were then computed independently within each cohort and were available for all final model-eligible samples. Signature-gene coverage was assessed across all training and validation datasets to document platform- and annotation-related differences and to identify dataset-specific missing genes. The number and identity of missing genes for each signature within each cohort are shown in Supplementary Fig. 2.

**Table 1:** Patient clinicopathologic characteristics and follow-up data across the four dataset groups

Characteristic	MetaGxBreast (N = 1997)	GEO (N = 321)	SBCC (N = 322)	SCAN-B (N = 2492)	*p* value	Cramér’s V
Age					< 0.001	0.192
< 40	92 (5%)	0 (0%)	11 (3%)	3 (0%)		
40–49	365 (18%)	18 (6%)	37 (12%)	102 (4%)		
50–64	702 (35%)	120 (37%)	119 (37%)	602 (24%)		
≥ 65	838 (42%)	183 (57%)	155 (48%)	1785 (72%)		
Tumour size					< 0.001	0.202
< 2 cm	828 (41%)	118 (37%)	158 (49%)	1745 (70%)		
2–5 cm	1109 (56%)	192 (60%)	156 (48%)	697 (28%)		
> 5 cm	60 (3%)	11 (3%)	8 (3%)	50 (2%)		
Grade					< 0.001	0.158
Grade I	377 (19%)	60 (19%)	89 (28%)	657 (26%)		
Grade II	985 (49%)	194 (60%)	177 (55%)	1524 (61%)		
Grade III	635 (32%)	67 (21%)	56 (17%)	311 (13%)		
Lymph Nodes					< 0.001	0.163
Negative	1395 (70%)	146 (45%)	238 (74%)	1901 (76%)		
Positive	602 (30%)	175 (55%)	84 (26%)	591 (24%)		
Treatment					< 0.001	0.377
No systemic therapy	953 (48%)	18 (6%)	116 (36%)	340 (14%)		
Endocrine therapy	1044 (52%)	303 (94%)	206 (64%)	2152 (86%)		
Median follow-up (years)	9.6	12.2	15.0	5.3		
Selected horizon (years)	15	15	15	10		
Events (N) (within horizon)	446	52	96	108		
Horizon‑evaluable event rate	57.7%	34.4%	46.4%	39.1%		

Values are shown as n (%) unless otherwise indicated. *p* values were calculated using Pearson’s chi-squared test for differences across dataset groups, and Cramér’s V is reported as the corresponding effect-size measure for categorical variables. Events and crude event rates are reported within the selected prediction horizon for each dataset group. For crude event rates, the denominator corresponds to patients evaluable at the selected horizon, rather than the full cohort size

### Feature selection and model training

Across all analyses, each feature demonstrated clear prognostic value on Kaplan–Meier curves (Supplementary Fig. 3) and remained significant in multivariable Cox models, confirming their independent contributions to risk prediction (Supplementary Fig. 4 and Supplementary Table 2). Assessment using Schoenfeld residual-based diagnostics indicated violation of proportional hazards assumption for GESs and CCRS (Supplementary Fig. 5), suggesting that their prognostic effect on DMFS may be time-dependent rather than constant over follow-up. Pairwise correlations among the three features were uniformly low (Supplementary Fig. 6), indicating minimal redundancy and supporting their use as complementary predictors within the modelling framework.

We developed a final clinicogenomic RSF model incorporating the cohort-percentile 21-gene RS, the 14-gene IgG mean gene-wise Z-score, and the CCRS. The 21-gene RS was selected as the genomic signature component because, among the evaluated GESs, it showed robust prognostic performance with the narrowest 95% CI in the training dataset (Supplementary Fig. 4). Feature-score distributions across the four dataset groups after harmonisation are shown in Supplementary Fig. 7.

Based on OOB performance, the final model was trained using 2000 trees with a maximum tree depth of 11, which represented the best-performing configuration within the evaluated hyperparameter grid and lay within the region where performance largely stabilised (Supplementary Fig. 8). Following the predefined hyperparameter rules, the number of variables considered at each split was set to 1 (*mtry* = *1*), corresponding to the rounded-down square root of the three input features. The minimum terminal node size was set to 40 (*min.node.size* = *40*), corresponding to the larger of 20 observations or 2% of the *MetaGxBreast* training sample size, rounded up.

### Model validation, performance evaluation, and explainability

**Fig. 2:**
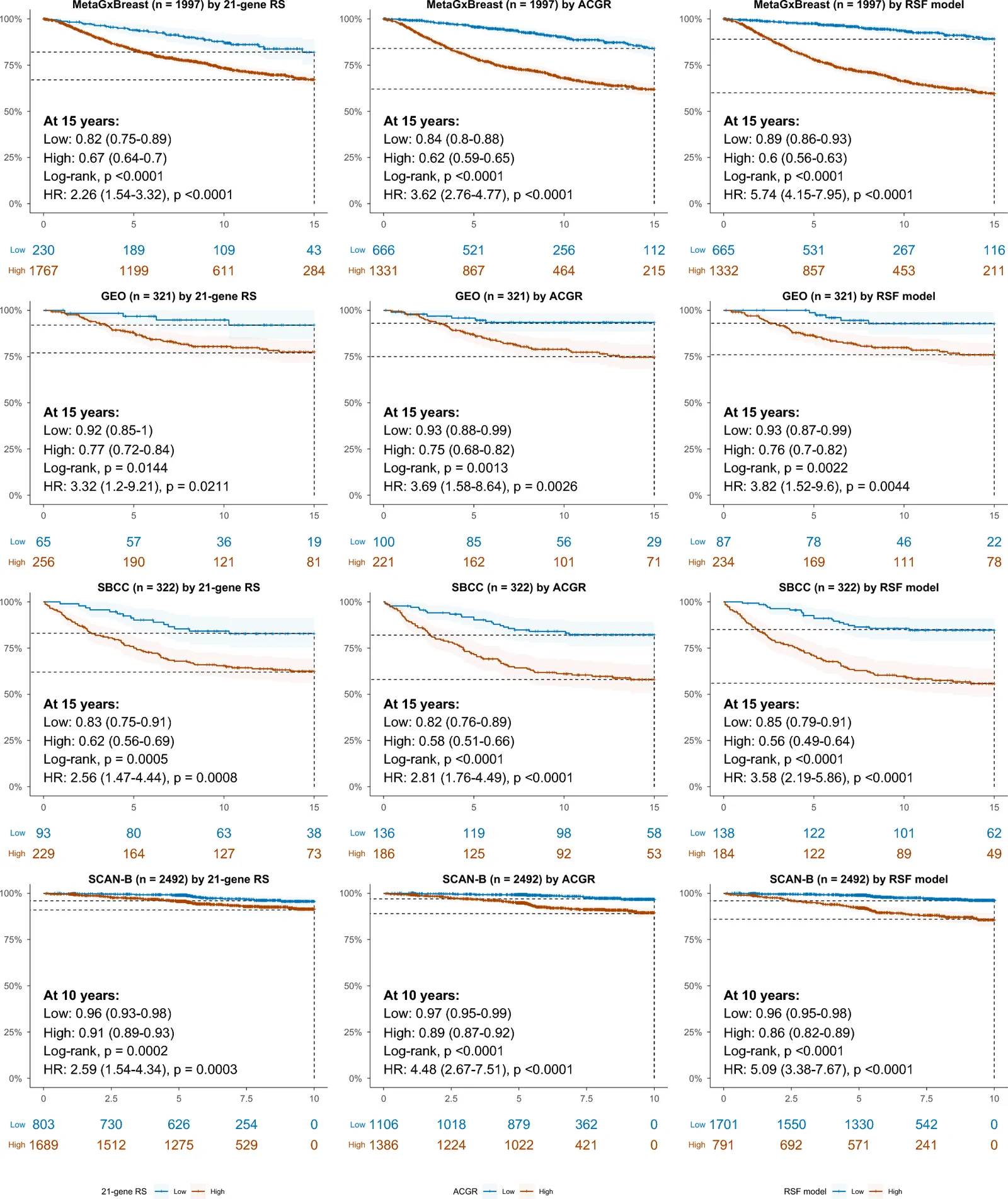
Risk stratification by the 21-gene RS, ACGR score, and RSF model across training and validation datasets. Kaplan–Meier curves for DMFS in low- and high-risk groups in the *MetaGxBreast* training dataset and the three external validation datasets (*GEO*, *SBCC*, *SCAN-B*), stratified by the 21-gene RS (left column), the ACGR score (middle column), and the RSF model combining the cohort-percentile 21-gene RS, the IgG signature, and the CCRS (right column). For the 21-gene RS, risk groups were defined using the default categories of the research-grade implementation, with the intermediate- and high-risk categories combined into a single high-risk group. For ACGR and the RSF model, the low/high cut-offs were the lower tertiles of their respective score distributions in the *MetaGxBreast* training dataset and were applied unchanged to all validation datasets. Low-risk groups are shown in blue and high-risk groups in dark orange; shaded bands are 95% CIs and plus symbols denote censored observations. Each panel is annotated with the Kaplan–Meier DMFS probability (95% CI) per risk group at the dataset-specific evaluation horizon (15 years for *MetaGxBreast*, *GEO* and *SBCC*; 10 years for *SCAN-B*), the log-rank *p* value, and the HR (95% CI) for high versus low risk. The number-at-risk table is shown below each plot at 0, 5, 10, and 15 years (0, 2.5, 5, 7.5, and 10 years for SCAN-B)

The model’s prognostic performance was first evaluated through risk-stratification analyses. For the standalone GES, the predefined risk stratification was retained. For ACGR and the RSF model, low-risk groups were defined using the predicted score lower-tertile cut-offs derived in the *MetaGxBreast* training dataset and applied unchanged to all validation datasets. Kaplan–Meier curves and risk group comparisons for each approach (the individual GES, the ACGR, and the RSF model) showed clear separation between predicted risk groups (Fig. 2). Compared with the standalone GES, the RSF model identified larger low-risk groups across all cohorts while maintaining high low-risk survival estimates, although the relative balance between low-risk group size and survival varied across validation datasets.

Across the validation datasets, all three approaches identified low-risk groups with favourable horizon-specific DMFS estimates. The standalone GES classified 20.2–32.2% of validation patients as low risk, with survival estimates ranging from 83 to 96%. ACGR increased the low-risk fraction to 31.2–44.4% while maintaining comparable survival estimates. The RSF model identified the largest low-risk groups in *SBCC* and *SCAN-B*, classifying 42.9% and 68.3% of patients as low risk, respectively, with survival estimates of 85% and 96%. In *GEO*, the RSF model classified 27.1% of patients as low risk, with survival comparable to ACGR. Detailed low-risk group sizes, corresponding proportions, and survival estimates for each dataset are provided in Supplementary Table 3.

To further assess performance, discrimination (C-index and iAUC), risk-stratification (ΔRMST between low- and high-risk groups), and prediction-error (Cox-recalibrated IBS and IPA) metrics were computed for the GES alone, the ACGR score, and the RSF model and are summarised in Table 2. Across datasets, the RSF model generally showed higher C-index and iAUC values and larger ΔRMST values than the standalone GES, whereas Cox-recalibrated IBS values were comparable or lower depending on the dataset. Paired bootstrap comparisons supported significant improvements of the RSF model over the GES in *MetaGxBreast*, *SBCC*, and *SCAN-B*, whereas differences in *GEO* were numerically favourable but not statistically supported (Supplementary Table 4). Compared with the ACGR score, the RSF model showed comparable performance. Calibration plots provided descriptive horizon-specific diagnostics (Supplementary Fig. 9). Because the GES and ACGR plots were Cox-recalibrated score-calibration diagnostics, whereas the RSF plot used native predicted risks, these plots were not interpreted as a formal three-way native calibration comparison. Overall, calibration varied across cohorts, reflecting differences in baseline risk, follow-up duration, and case mix; therefore, IBS values were used as the primary quantitative prediction-error summary.

**Table 2:** Comparative performance of the GES, ACGR score, and RSF model across training and validation datasets

Dataset	N	Horizon (years)	Discrimination measures					
			C-index			iAUC		
			GES	ACGR	RSF model	GES	ACGR	RSF model
MetaGxBreast	1997	15	0.645 (0.610–0.677)	0.680 (0.651–0.709)	0.724 (0.697–0.751)	0.664 (0.622–0.704)	0.710 (0.676–0.742)	0.754 (0.720–0.785)
GEO	321	15	0.633 (0.548–0.715)	0.673 (0.575–0.763)	0.668 (0.573–0.763)	0.606 (0.456–0.762)	0.644 (0.378–0.863)	0.636 (0.410–0.842)
SBCC	322	15	0.650 (0.592–0.712)	0.694 (0.639–0.754)	0.708 (0.654–0.761)	0.689 (0.622–0.763)	0.740 (0.675–0.809)	0.752 (0.698–0.819)
SCAN-B	2492	10	0.724 (0.668–0.773)	0.752 (0.699–0.799)	0.778 (0.731–0.823)	0.714 (0.650–0.772)	0.760 (0.705–0.814)	0.778 (0.723–0.828)

Dataset	N	Horizon (years)	ΔRMST (years), pooled auxiliary			IBS, Cox-recalibrated		
			GES	ACGR	RSF model	GES	ACGR	RSF model
MetaGxBreast	1997	15	1.63	2.58	3.08	0.131 (0.113–0.137)	0.126 (0.108–0.133)	0.119 (0.102–0.126)
GEO	321	15	1.51	1.63	1.73	0.073 (0.047–0.092)	0.071 (0.046–0.090)	0.072 (0.047–0.091)
SBCC	322	15	2.40	2.74	3.29	0.148 (0.119–0.161)	0.142 (0.113–0.154)	0.142 (0.113–0.154)
SCAN-B	2492	10	0.26	0.42	0.61	0.031 (0.026–0.037)	0.030 (0.025–0.036)	0.030 (0.025–0.035)

Dataset	N	Horizon (years)	KM reference IBS			IPA vs KM		
						GES	ACGR	RSF model
MetaGxBreast	1997	15	0.139 (0.119–0.145)			0.055 (0.014–0.083)	0.090 (0.041–0.116)	0.139 (0.083–0.170)
GEO	321	15	0.076 (0.049–0.097)			0.039 (− 0.018–0.105)	0.060 (− 0.017–0.143)	0.044 (− 0.019–0.115)
SBCC	322	15	0.159 (0.130–0.174)			0.070 (0.027–0.134)	0.109 (0.055–0.191)	0.110 (0.055–0.194)
SCAN-B	2492	10	0.032 (0.026–0.038)			0.025 (0.012–0.045)	0.051 (0.026–0.085)	0.055 (0.030–0.087)

Performance was evaluated at 15 years for *MetaGxBreast*, *GEO*, and *SBCC*, and at 10 years for *SCAN-B*. Discrimination was assessed using C-index and iAUC, risk separation using ΔRMST, and prediction error using Cox-recalibrated IBS. IPA was calculated relative to the Kaplan-Meier reference model. Values in parentheses indicate 95% CIs. Higher C-index, iAUC, ΔRMST, and IPA indicate better performance, whereas lower IBS indicates lower prediction error.

*N* denotes the overall analysis-dataset sample size. For iAUC, IBS, and IPA, only eligible original cohorts with at least 20 evaluable patients and at least five events contributed; effective sample sizes may therefore be smaller.

Because ΔRMST is horizon-dependent, the SCAN-B ΔRMST estimate was calculated over a shorter 10-year window and should not be directly compared with the 15-year ΔRMST estimates from the other datasets.

In addition, comparison of risk stratification performance against the remaining GESs not included in model development showed that the RSF model identified larger low-risk groups than the 12-gene MS and 70-gene signature across datasets, and the largest low-risk group overall in *SCAN-B*, while maintaining comparable or improved survival estimates relative to the alternative GESs (Supplementary Fig. 10).

To contextualise model development within the heterogeneous training data, we further examined the composition and outcome distributions of the *MetaGxBreast* dataset. The *MetaGxBreast* training compendium comprised 1997 patients from 12 original cohorts, with 446 DMFS events (Supplementary Table 5). Cohort sizes, follow-up and event burden varied substantially. Cohort-specific Kaplan–Meier curves demonstrated visible differences in DMFS distributions, with 5-year DMFS estimates ranging from 73.0 to 98.8% and 10-year estimates ranging from 64.8 to 96.6% among cohorts with sufficient follow-up (Supplementary Fig. 11). Fifteen-year estimates were available for fewer cohorts and should be interpreted cautiously because several original cohorts had limited numbers at risk at later time points.

The proportion of patients classified as low risk by the RSF model differed across datasets, ranging from 27.1% in *GEO* to 68.3% in *SCAN-B* (Supplementary Table 6). This variation was accompanied by differences in the distribution of model-predicted risk, with *SCAN-B* showing a substantially lower median predicted risk than the other datasets (0.206 versus 0.274–0.306). *SCAN-B* also had a more favourable clinicopathologic composition, including higher proportions of patients with tumours < 2 cm, grade I disease, node-negative disease, and endocrine therapy use. Although baseline KM survival at the evaluation horizon was highest in *SCAN-B*, the dataset-level rank correlation between baseline DMFS and the RSF low-risk fraction was weak and purely descriptive (Spearman ρ = 0.20; n = 4). These findings suggest that the high RSF-defined low-risk proportion in *SCAN-B* should be interpreted in the context of its lower-risk case mix, shorter evaluation horizon, and lower predicted-risk distribution, rather than as an isolated model effect.

Because *SCAN-B* had the most favourable clinicopathologic composition, lowest median predicted RSF risk, and largest RSF-defined low-risk fraction, we performed dedicated subgroup analyses within this dataset. Applying the training-derived RSF cut-off unchanged, the model retained prognostic separation across both age and tumour-size strata (Supplementary Fig. 12). RSF C-index estimates ranged from 0.700 to 0.787 across subgroups, although 10-year AUC estimates varied, particularly by age (0.572 in patients aged < 65 years versus 0.729 in those aged ≥ 65 years); the estimate in the younger subgroup should be interpreted in the context of its limited number of events (n = 24). Interaction analyses did not show evidence that the association between RSF risk and outcome differed by age, either when RSF risk was modelled as a dichotomised low/high group (LRT, *p* = 0.364) or as a continuous predicted-risk score (*p* = 0.236). Similarly, there was no evidence of interaction by tumour size using either the dichotomised RSF group (*p* = 0.398) or continuous RSF risk (*p* = 0.651; Supplementary Table 7). Thus, *SCAN-B*’s high low-risk fraction appears consistent with its lower-risk case mix, while the RSF model preserved risk separation within clinically relevant subgroups.

Because the final study population included both untreated and endocrine-treated patients, we further evaluated the RSF model within *SCAN-B* by treatment status. Using the same training-derived cut-off without retraining or recalibration, the RSF model stratified outcomes in both treatment-defined subgroups (Supplementary Fig. 13). Discrimination was maintained in both subsets, with RSF C-index values of 0.869 and 0.767 and 10-year iAUC values of 0.859 and 0.774 in untreated and endocrine-treated patients, respectively (Supplementary Table 8). Interaction analyses did not show evidence that the RSF–outcome association differed by treatment status, either for dichotomised RSF group (LRT, *p* = 0.348) or continuous RSF-predicted risk (*p* = 0.251). These findings support that the RSF model retained prognostic stratification across treatment-defined *SCAN-B* subsets, while the untreated subgroup had limited event numbers and wider uncertainty.

To further benchmark the RSF model against an established clinicogenomic risk-stratification paradigm, we compared its performance with a transparent *RSClin*-analogous Cox comparator in node-negative patients. This comparator integrated the 21-gene RS with age, tumour size, and histological grade, reflecting the published *RSClin* covariate structure while not reproducing the commercial *RSClin* tool. At the common 10-year evaluation horizon, the *RSClin*-analogous comparator improved discrimination over the 21-gene RS alone in all four node-negative subsets. The RSF model showed higher discrimination than the *RSClin*-analogous comparator in *MetaGxBreast*, *SBCC*, and *SCAN-B* and nearly identical performance in *GEO* (Supplementary Table 9A). Adding IgG improved model fit in the *MetaGxBreast* node-negative training subset (LRT, *p* < 0.0001; Supplementary Table 9B) and increased the 10-year C-index in *MetaGxBreast*, *GEO*, and *SBCC*, but not in *SCAN-B* (Supplementary Table 9A). Thus, the RSF model performed generally comparably to the base *RSClin*-analogous clinicogenomic benchmark, whereas its performance relative to the IgG-extended comparator and the incremental value of IgG were cohort-dependent.

**Fig. 3:**
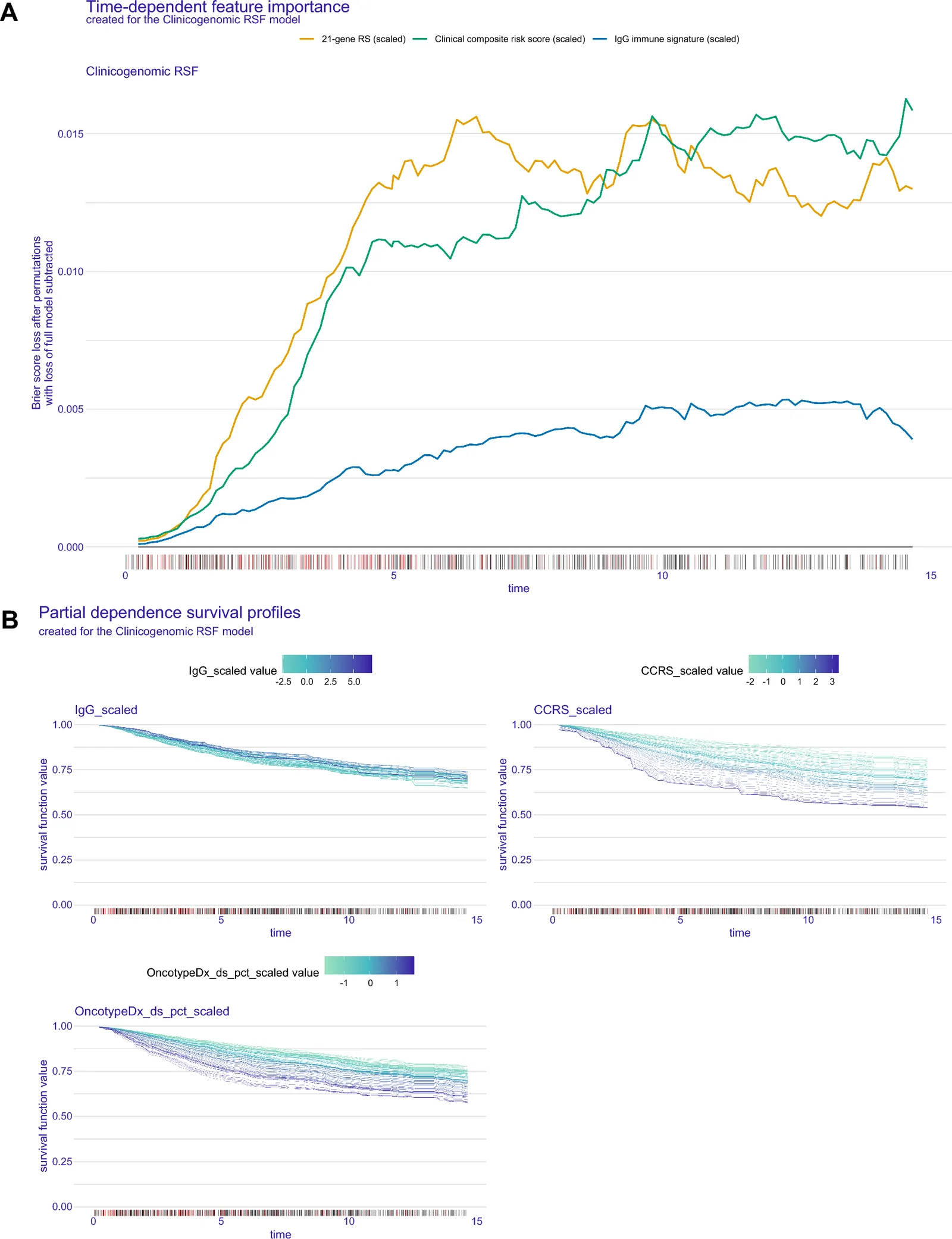
Explainability analyses of the trained RSF model in the *MetaGxBreast* training dataset. **A** Time-dependent permutation-based feature importance, quantified as the increase in prediction error (Brier score) after permuting each model input relative to the unpermuted model. Larger values indicate a greater contribution to model performance. **B** Time-dependent partial-dependence survival profiles, showing how the predicted survival function varied across values of each feature after averaging predictions over the empirical distribution of the remaining model features. The three model inputs were the within-cohort percentile rank of the 21-gene RS, the IgG signature, and the CCRS; all three were standardised using parameters estimated in the training dataset

To characterise model behaviour and explainability, we generated model-level performance diagnostics, permutation-based feature-importance analyses, SurvSHAP(t) summaries, and partial-dependence plots. Model-level diagnostics showed the time-resolved Brier score, cumulative/dynamic AUC, and deviance residual patterns, supporting the overall stability of the fitted RSF model (Supplementary Fig. 14). Feature-importance analyses consistently identified the CCRS and the 21-gene RS as the dominant contributors to prognostic performance over follow-up time, whereas IgG showed a smaller but more stable contribution (Fig. 3A and Supplementary Fig. 15A). This pattern was further supported by SurvSHAP(t), which showed the largest overall and time-varying contributions for the CCRS and the 21-gene RS, with a weaker but persistent contribution from IgG (Supplementary Fig. 15B). Partial-dependence plots showed directionally consistent feature effects: increasing CCRS or 21-gene RS shifted predicted survival probabilities downward, whereas higher IgG values were associated with a modest upward shift in predicted survival (Fig. 3B). Together, these analyses indicate that the RSF model primarily captures clinicopathologic, proliferation-related, and estrogen receptor signalling-related risk, while retaining a smaller but stable immune-related prognostic contribution over time.

## Discussion

This study examined whether a clinicogenomic survival machine learning model could improve prognostic stratification beyond established GESs in ER-positive/HER2-negative early breast cancer. Across datasets, the RSF model generally improved prognostic performance compared with the standalone GES and showed performance comparable to ACGR, although results varied across cohorts and metrics. Importantly, performance was maintained across heterogeneous expression-profiling technologies, including both microarray and RNA-seq data, supporting the technical robustness of the model. The most clinically relevant finding was that the RSF model generally identified larger low-risk groups than the standalone GES while preserving comparable or improved survival estimates. This suggests that the model may improve risk stratification primarily by expanding the group of patients classified as low risk without materially compromising outcome separation. Overall, these findings support the value of integrating established GESs with immune-related and clinicopathologic information, while emphasising that the added value of machine learning over simpler composite scores is cohort- and metric-dependent.

The different proportions of predicted low-risk patients across cohorts likely reflect differences in case mix rather than instability of the model. This was most evident in *SCAN-B*, where the large low-risk fraction is consistent with its lower-risk clinical composition. Stratified analyses showed no significant interaction between RSF risk group and age or tumour size, supporting preserved risk separation within *SCAN-B* subgroups. Nevertheless, the absolute low-risk fraction in *SCAN-B* should be interpreted cautiously and may not be directly generalisable to higher-risk populations.

The present framework extends existing multimodal prognostic approaches, such as *RSClin*, which integrate the 21-gene RS with clinicopathologic variables to improve individualised risk assessment. In the node-negative benchmark analysis, adding the IgG signature to the *RSClin*-analogous comparator improved discrimination in several datasets, although this contribution was not consistent across cohorts. The RSF model showed the clearest advantage in *MetaGxBreast* and remained broadly comparable with the IgG-extended *RSClin*-analogous benchmark in *SBCC* and *SCAN-B*, whereas the IgG-extended comparator showed higher discrimination in *GEO*. These findings suggest that immune-related information may provide prognostic value beyond established genomic and clinicopathologic factors, but that both its incremental contribution and the potential advantage of nonlinear modelling depend on the study population. Accordingly, the main potential value of the RSF model lies not only in discrimination, but also in its ability to identify larger low-risk groups while maintaining comparable or improved survival estimates.

Explainability analyses are essential for clinical translation, as they help clinicians understand how individual predictors contribute to risk estimation and can also reveal the temporal behaviour of the model. In our study, the time-dependent feature-importance analysis showed that the 21-gene RS and CCRS had the strongest prognostic influence, increasing during the first years after diagnosis and peaking around the first decade. This pattern is consistent with the established clinical behaviour of ER-positive/HER2-negative breast cancer, where recurrence risk can persist over prolonged follow-up [41], but the relative contribution of baseline proliferation- and clinicopathology-related risk may vary over time. In contrast, the IgG immune component had a smaller but more sustained contribution throughout follow-up. This supports the concept that immune activity contributes to prognosis in ER-positive breast cancer, although the immunobiology of ER-positive disease remains less well characterised than in other types [42]. The IgG signature captures a focused subset of humoral and lymphocyte-related immune activity, reflecting genes involved in lymphocyte maturation, activation, chemotaxis, and immunoglobulin production, and has been associated with improved overall survival among breast cancer patients without documented recurrence [43]. Consistent with these external data, IgG was also independently prognostic for DMFS in the *MetaGxBreast* training dataset in our analysis. As this represents only one component of the broader and more complex immune landscape, its prognostic contribution may be distinct but comparatively modest. Importantly, ER-positive/HER2-negative tumours represent a biologically heterogeneous spectrum rather than a single uniform entity [44], and immune infiltration is unlikely to contribute equally across all luminal cancers. Consistent with this, adding IgG to the *RSClin*-analogous comparator improved discrimination in *MetaGxBreast*, *GEO*, and *SBCC*, but not in node-negative *SCAN-B*. These findings suggest that IgG provides complementary prognostic information in selected contexts, but that its incremental value may depend on cohort composition, baseline clinical risk, and underlying tumour biology.

This study has several limitations that should be considered when interpreting the potential clinical relevance of the RSF framework. The model integrates routinely available clinicopathologic variables with gene expression features, supporting the feasibility of a multimodal prognostic approach. However, the present findings remain exploratory and should not be interpreted as directly practice-changing. Although the study focused on ER-positive/HER2-negative tumours, the analysis was not restricted to the exact intended-use populations of current commercial assays. Moreover, the GESs were calculated using research-grade transcriptomic implementations rather than clinically generated commercial assay results. This distinction is important because discordance has been reported not only between research-based implementations and commercial assay outputs, but also across approved commercial assays themselves [45, 46], which may impair their clinical application [47]. In addition, the RSF model used a cohort-relative percentile transformation of the research-grade 21-gene RS to reduce cross-cohort scale differences. Therefore, neither the model inputs nor the resulting risk groups should be interpreted as equivalent to clinically validated assay outputs or risk categories used for adjuvant treatment decisions. The model-defined low- and high-risk groups represent study-specific prognostic strata and should not be used to infer chemotherapy eligibility or de-escalation in individual patients. Rather, a potentially clinically relevant observation is that the RSF model identified larger groups of patients with favourable long-term outcomes compared with standalone research-grade GESs, while broadly preserving outcome separation. This suggests possible future relevance for treatment de-escalation evidence, particularly as modern endocrine therapies continue to improve outcomes and may further reduce the absolute recurrence risk in patients with favourable baseline prognosis. However, such use would require prospective validation in clinically appropriate populations using standardised assay outputs.

Moreover, the analysis relied on a large collection of clinically and technically heterogeneous datasets generated across different institutions, expression profiling platforms, and historical treatment periods. This diversity increases the breadth of external evaluation, but it also introduces variation that may influence model performance and patient outcomes [48]. Technical sources of heterogeneity include batch effects, laboratory and platform differences, and inconsistent normalisation and preprocessing pipelines. Although harmonisation procedures were implemented, some loss or distortion of biological signal is unavoidable. Limitations related to gene annotation must also be acknowledged, particularly for older microarray platforms where probe mappings may be incomplete or outdated, potentially affecting gene capture and signature completeness. Clinical heterogeneity represents an additional source of variation. Patients were treated at multiple centres, under differing clinical routines, and across periods spanning more than four decades. The duration and administration of endocrine therapy also varied considerably, which may influence long-term outcomes.

Survival endpoints were also not fully uniform across validation datasets, reflecting differences in endpoint availability and cohort-specific follow-up annotation. Although DMFS was used wherever available, closely related recurrence endpoints were required for selected cohorts, including DRFS in two *SBCC* cohorts, RFS in the small *GSE26338 GEO* cohort, and DRFi in *SCAN-B*. *SCAN-B* was also evaluated at a 10-year horizon because longer-term outcome data were limited. Importantly, the *GSE26338* *GEO* cohort contributed just 18 patients and one event, making a material influence on the overall *GEO* estimates unlikely. Nevertheless, these endpoints are not identical, and residual endpoint heterogeneity may have contributed to differences in performance estimates across cohorts.

In addition, receptor-status harmonisation may have introduced some misclassification. ER status was approximated from *ESR1* expression in *GSE158309*, and HER2 status was inferred from *ERBB2* expression when clinical HER2 annotation was unavailable. Even among datasets with IHC-based receptor annotation, differences in assay methods and positivity thresholds across institutions and time periods may have contributed additional classification variability.

Our findings suggest that integrating established research-grade GESs with immune-related and clinicopathologic information can improve prognostic stratification beyond individual GESs alone. The RSF model identified larger low-risk patient groups than the standalone GES while maintaining comparable or higher survival estimates, with modest and generally directionally consistent improvements in discrimination and prediction error across validation cohorts. Feature-level explainability analyses further supported the biological plausibility of the model by highlighting time-dependent contributions of genomic, immune-related, and clinical variables. Together, these findings support the potential value of an explainable clinicogenomic modelling framework, although prospective validation will be required to determine its clinical applicability.

## Supplementary Information

Supplementary Material 1

## Data Availability

Publicly available data analysed in this study were obtained from the *MetaGxBreast* R package, the Gene Expression Omnibus (GEO) repository, *SCAN-B*, and previously published studies, as indicated in the manuscript and Supplementary Material. The source datasets are not redistributed and should be obtained from their original repositories. Patient-level data from the institutional *SBCC* dataset are not publicly available because of ethical and legal restrictions but may be made available following a formal request to the corresponding author and subject to the necessary ethical and institutional approvals. The code for the publicly reproducible analyses is available in the GitHub repository https://github.com/foukakis-lab/rsf-bca-prognostication. A permanent, versioned archive of the public analysis code and results is available through Zenodo (version 1.0.0; https://doi.org/10.5281/zenodo.22647156). The archived release includes the analysis and helper scripts, the public data preparation workflow and instructions, the locked R package environment, and all publicly shareable aggregate results generated from the *MetaGxBreast*, *GEO*, and *SCAN-B* datasets. Patient-level *SBCC* data and the corresponding analyses and results are not included in the public archive.
