# Supplementary material for: Development and validation of a machine learning clinicogenomic model to improve prognostic stratification in ER-positive/HER2-negative early breast cancer
Source: Breast Cancer Res. 2026 Sep 16;28:156. doi: 10.1186/s13058-026-02388-4 (PMC13584486; doi:10.1186/s13058-026-02388-4)
Supplement: Supplementary file 1 [file 13058_2026_2388_MOESM1_ESM.docx]

# Supplementary Methods

**Clinical data enrichment and harmonisation of study cohorts**

To maximise the number of samples with complete clinicopathologic and follow-up information and thereby enable model development on the largest possible dataset, the *MetaGxBreast* dataset was systematically enriched using external public metadata and annotation resources. When essential clinical information was missing or incomplete, variables were retrieved, re-derived, or harmonised from original study metadata, *cBioPortal* (<https://www.cbioportal.org/>), GDC-derived TCGA clinical files (<https://portal.gdc.cancer.gov/>), or published supplementary resources. All added variables were processed using cohort-specific rules and standardised definitions to ensure consistency across cohorts while preserving the original structure of each cohort.

For cases in which measured tumour size was not available but American Joint Committee on Cancer (AJCC) T stage was reported, tumour size category was derived from the T category ^1^. T1, T2, and T3 tumours were mapped to <2 cm, 2–5 cm, and >5 cm, respectively. T4 tumours were set to missing for tumour-size category, because T4 denotes direct extension to the chest wall and/or skin irrespective of tumour size.

For the LUND cohort, clinical metadata were enriched using GSE31863 ^2^. Histologic grade was derived according to the Nottingham Combined Histologic Grade system, using tubule formation, nuclear pleomorphism, and mitotic count. DMFS time and event status were supplemented using reported time to distant recurrence and follow-up time. In addition, the LUND expression dataset was replaced with the GSE31863 expression matrix, using *MetaGxBreast*-compatible sample identifiers, and probe annotation was updated by mapping *Ensembl* identifiers to gene symbols and Entrez Gene IDs.

For the METABRIC cohort, expression-sample phenotypes were updated using matched *cBioPortal* clinical data (<https://www.cbioportal.org/study/clinicalData?id=brca_metabric>) and a published patient-level recurrence-history file ^3^. Histologic grade and tumour size were supplemented from *cBioPortal* clinical annotations. DMFS-like information was updated using the published METABRIC recurrence-history data.

For the TCGA-BRCA cohort, sample identifiers were first harmonised to ensure consistency between expression and clinical tables. Disease-free survival information, tissue-source site, and AJCC T stage were obtained from the Breast Invasive Carcinoma TCGA PanCancer Atlas clinical data (<https://www.cbioportal.org/study/clinicalData?id=brca_tcga_pan_can_atlas_2018>). Because measured continuous tumour size was not available, tumour size category was derived from AJCC T stage as described above. Lymph node status was derived from GDC clinical AJCC N-stage annotations. Histologic grade was derived from published TCGA histologic annotations using the Nottingham Combined Histologic Grade system based on tubule formation, nuclear pleomorphism, and mitotic count ^4^. Missing TCGA HER2 status was conservatively updated using definitive BCR IHC status, or FISH status when IHC was unavailable or non-definitive. Treatment status was curated from TCGA BCR drug-level records and GDC pharmaceutical-therapy annotations, using drug-name and therapy-type evidence to distinguish endocrine-only treatment, untreated cases, and cases receiving chemotherapy, targeted therapy, mixed, or non-classifiable systemic treatment.

# Supplementary Tables

**Supplementary Table 1: Summary of dataset groups and individual study cohorts.**

For each cohort included in the training and validation datasets, the table reports the contributing institution(s) and region(s), original sample size, final model-eligible sample size, gene expression platform or technology, years of sampling, and primary reference. Dataset-group headers indicate the final number of samples contributing to each analysis dataset.

| **Dataset** | **Institution(s) and region(s)** | **Total sample size** | **Final sample size** | **Platform or technology** | **Years of sampling** | **Reference**  **(PMID)** |
| --- | --- | --- | --- | --- | --- | --- |
| **MetaGxBreast (n = 1,997)** | | | | | | |
| **CAL** | - UC San Francisco (UCSF), USA - California Pacific Medical Center (CPMC), USA | 118 | 29 | Affymetrix High Throughput Array (HTA) GeneChip | 1989 - 1997 | 17157792 |
| **DFHCC** | Dana-Farber/Harvard Cancer Center (DF/HCC), Massachusetts, USA | 115 | 19 | Affymetrix Human Genome U133 Plus 2.0 Array | Not specified | 20098429 |
| **LUND** | Southern, Western, and Stockholm healthcare regions, Sweden | 143 | 84 | SWEGENE Human v2.1.1 55K | 1981 - 1997 | 18430221 |
| **MAINZ** | Johannes Gutenberg University Mainz, Germany | 200 | 146 | Affymetrix Human Genome U133A Array | 1988 - 1998 | 18593943 |
| **NKI** | Netherlands Cancer Institute (NKI), Netherlands | 337 | 152 | Rosetta Inpharmatics | 1984 - 1995 | 12490681 |
| **TRANSBIG** | - Institut Gustave Roussy (IGR), Villejuif, France - Centre René Huguenin (CRH), Saint-Cloud, France - Guy's Hospital (GH), London, UK - John Radcliffe Hospital (JRH), Oxford, UK | 198 | 79 | Affymetrix Human Genome U133A Array | 1980 - 1998 | 17545524 |
| **UCSF** | - UC San Francisco (UCSF), USA - California Pacific Medical Center (CPMC), USA | 161 | 15 | Custom spotted cDNA microarray | Not specified | 17428335 |
| **UNT** | John Radcliffe Hospital (JRH), Oxford, UK | 133 | 19 | Affymetrix Human Genome U133A Array | Not specified | 16478745 |
| **VDX** | Erasmus Medical Center (EMC), Rotterdam, Netherlands | 344 | 119 | Affymetrix Human Genome U133A Array | 1980 - 1995 | 15721472 |
| **METABRIC** | - UK - Canada | 2114 | 1100 | HumanHT-12 v3.0 Gene Expression BeadChip | 1977 - 2005 | 22522925 |
| **TCGA** | - USA - Poland - Germany | 1073 | 183 | Agilent 244K whole genome custom Microarray | 1988 - 2011 | 23000897 |
| **GSE58644** | McGill University and Génome Québec Innovation Centre (MUGQIC), Canada | 318 | 52 | Affymetrix Human Gene 1.0 ST Array | 1999 - 2012 | 25284793 |
| **GEO (n = 321)** | | | | | | |
| **GSE6532 (GPL570)** | Guy's Hospital (GH), London, UK | 87 | 61 | Affymetrix Human Genome U133 Plus 2.0 Array | 1980 - 1995 | 18498629 |
| **GSE6532 (GPL96)** | John Radcliffe Hospital (JRH), Oxford, UK | 327 | 42 | Affymetrix Human Genome U133A Array | 1980 - 1995 | 18498629 |
| **GSE9195** | Guy's Hospital (GH), London, UK | 77 | 56 | Affymetrix Human Genome U133 Plus 2.0 Array | Not specified | 18498629 |
| **GSE26338** | - University of North Carolina at Chapel Hill, USA - University of Utah, USA - Thomas Jefferson University, USA - University of Chicago, USA | 414 | 18 | Agilent Human 1A Oligo UNC custom Microarray | Not specified | 21671017 and 17663798 |
| **GSE45255** | - Institut Jules Bordet, Brussels, Belgium - John Radcliffe Hospital (JRH), Oxford, UK - National University Hospital, Singapore | 139 | 25 | Affymetrix Human Genome U133A Array | 1990 - 2002 | 23618380 |
| **GSE158309** | University Medical Center Mainz, Germany | 461 | 119 | Affymetrix Human Genome U133A Array | 1986 - 2000 | 33003293 |
| **SBCC (n = 322)** | | | | | | |
| **Swedish Breast Cancer Cohort 1** | Stockholm Region, Sweden | 564 | 105 | Rosetta / Merck Human RSTA Custom Affymetrix 2.0 microarray | 1997 - 2005 | 28972043 |
| **Swedish Breast Cancer Cohort 2** | Karolinska Hospital, Sweden | 159 | 64 | Affymetrix Human Genome U133A Array | 1994 - 1996 | 16280042 |
| **Swedish Breast Cancer Cohort 3** | Uppsala University Hospital, Sweden | 253 | 153 | Affymetrix Human Genome U133A Array | 1987 - 1989 |  |
| **SCAN-B (n = 2,492)** | | | | | | |
| **SCAN-B** | Southern Healthcare Region, Sweden | 9,206 | 2,492 | RNA-seq | 2010 - 2018 | 35974007 |

**Supplementary Table 2**: **Multivariable Cox associations of gene expression signatures, IgG, and CCRS with DMFS in the MetaGxBreast training dataset.**

Separate Cox proportional hazards models were fitted for each gene expression signature combined with IgG and CCRS. Hazard ratios (HRs), 95% confidence intervals (CIs), and p values are shown for each model term. Continuous predictors were entered as standardised variables; therefore, HRs correspond to a one-standard-deviation increase. HR values above 1 indicate higher risk, whereas HR values below 1 indicate lower risk.

| **Model** | **Term** | **HR** | **95% CI  lower** | **95% CI upper** | **p value** |
| --- | --- | --- | --- | --- | --- |
| 21-gene RS + IgG + CCRS | 21-gene RS | 1.48 | 1.35 | 1.63 | <0.0001 |
| 21-gene RS + IgG + CCRS | IgG | 0.82 | 0.75 | 0.91 | <0.0001 |
| 21-gene RS + IgG + CCRS | CCRS | 1.76 | 1.58 | 1.95 | <0.0001 |
| ROR-S + IgG + CCRS | ROR-S | 1.49 | 1.34 | 1.65 | <0.0001 |
| ROR-S + IgG + CCRS | IgG | 0.84 | 0.77 | 0.92 | 0.0002 |
| ROR-S + IgG + CCRS | CCRS | 1.62 | 1.45 | 1.81 | <0.0001 |
| 12-gene MS + IgG + CCRS | 12-gene MS | 1.55 | 1.38 | 1.74 | <0.0001 |
| 12-gene MS + IgG + CCRS | IgG | 0.86 | 0.78 | 0.94 | 0.001 |
| 12-gene MS + IgG + CCRS | CCRS | 1.64 | 1.47 | 1.83 | <0.0001 |
| 70-gene signature + IgG + CCRS | 70-gene signature | 1.58 | 1.41 | 1.77 | <0.0001 |
| 70-gene signature + IgG + CCRS | IgG | 0.83 | 0.76 | 0.92 | 0.0002 |
| 70-gene signature + IgG + CCRS | CCRS | 1.61 | 1.44 | 1.80 | <0.0001 |

**Supplementary Table 3**: **Low-risk group size and survival estimates for the GES, ACGR score, and RSF model.**

Low-risk group sizes, proportions, and Kaplan–Meier survival estimates are shown at the selected prediction horizon for each dataset: 15 years for *MetaGxBreast*, *GEO*, and *SBCC*, and 10 years for *SCAN-B*. The GES corresponds to the research-grade 21-gene RS. The ACGR score is the average of the cohort-percentile 21-gene RS, inverted IgG score, and CCRS. The RSF model incorporates the cohort-percentile 21-gene RS, IgG, and CCRS, with CCRS summarising the clinicopathologic variables. Values are shown as n, percentage, and survival estimate with 95% confidence interval.

| **Dataset** | **GES** | | | **ACGR** | | | **RSF model** | | |
| --- | --- | --- | --- | --- | --- | --- | --- | --- | --- |
|  | **n** | **n%** | **KM DMFS survival  (95% CI)** | **n** | **n%** | **KM DMFS survival (95% CI)** | **n** | **n%** | **KM DMFS survival  (95% CI)** |
| **MetaGxBreast** | 230 | 11.5 | 0.82  (0.75–0.89) | 666 | 33.4 | 0.84  (0.80–0.88) | 665 | 33.3 | 0.89  (0.86–0.93) |
| **GEO** | 65 | 20.2 | 0.92  (0.85–1.00) | 100 | 31.2 | 0.93  (0.88–0.99) | 87 | 27.1 | 0.93 (0.87–0.99) |
| **SBCC** | 93 | 28.9 | 0.83  (0.75–0.91) | 136 | 42.2 | 0.82  (0.76–0.89) | 138 | 42.9 | 0.85  (0.79–0.91) |
| **SCAN-B** | 803 | 32.2 | 0.96  (0.93–0.98) | 1106 | 44.4 | 0.97  (0.95–0.99) | 1701 | 68.3 | 0.96  (0.95–0.98) |

**Supplementary Table 4**: **Paired bootstrap comparisons of RSF model performance against comparator models.**

Values show paired bootstrap mean differences with 95% confidence intervals and two-sided empirical p values. For C-index and iAUC, differences are calculated as RSF minus comparator; positive values favour the RSF model. For IBS, differences are calculated as comparator minus RSF; positive values indicate lower prediction error for the RSF model.

| **Cohort** | **Comparator** | **ΔC-index  (95% CI)** | **p value** | **ΔiAUC  (95% CI)** | **p value** | **ΔIBS  (95% CI)** | **p value** |
| --- | --- | --- | --- | --- | --- | --- | --- |
| **MetaGxBreast** | GES | 0.079  (0.056–0.100) | <0.001 | 0.090  (0.064–0.116) | <0.001 | 0.0107  (0.0076–0.0144) | <0.001 |
| **MetaGxBreast** | ACGR | 0.043  (0.027–0.060) | <0.001 | 0.043  (0.025–0.061) | <0.001 | 0.0063  (0.0038–0.0089) | <0.001 |
| **GEO** | GES | 0.034  (−0.025–0.096) | 0.238 | 0.027  (-0.073–0.133) | 0.587 | 0.0006  (−0.0036–0.0050) | 0.782 |
| **GEO** | ACGR | −0.004 (−0.041–0.032) | 0.816 | −0.004  (-0.059–0.055) | 0.858 | −0.0009  (−0.0041–0.0020) | 0.510 |
| **SBCC** | GES | 0.058  (0.018–0.104) | 0.010 | 0.066  (0.020–0.115) | 0.008 | 0.0065  (0.0002–0.0143) | 0.042 |
| **SBCC** | ACGR | 0.014  (−0.018–0.049) | 0.418 | 0.016  (-0.021–0.059) | 0.414 | 0.0002  (−0.0049–0.0051) | 0.912 |
| **SCAN-B** | GES | 0.054  (0.022–0.085) | 0.002 | 0.065  (0.026–0.102) | 0.006 | 0.0010  (0.0003–0.0018) | <0.001 |
| **SCAN-B** | ACGR | 0.026  (0.005–0.052) | 0.004 | 0.018  (-0.001–0.041) | 0.072 | 0.0001  (−0.0004–0.0006) | 0.614 |

**Supplementary Table 5**: **Cohort-specific follow-up and event summary for the *MetaGxBreast* training cohorts.**

For each cohort, the table reports the number of model-eligible patients, number of events, crude event rate, median and maximum follow-up duration, and Kaplan–Meier estimated survival probabilities at 5, 10, and 15 years. Event rate was calculated as the number of observed events divided by the number of patients. The -- symbol indicates that the Kaplan–Meier estimate was not available at the corresponding time point, usually because follow-up was insufficient or no patients remained at risk.

| **Cohort** | **Patients (n)** | **Events  (n)** | **Crude event rate** | **Median follow-up (years)** | **Maximum follow-up (years)** | **5-year  KM DMFS survival** | **10-year KM DMFS survival** | **15-year KM DMFS survival** |
| --- | --- | --- | --- | --- | --- | --- | --- | --- |
| **METABRIC** | 1100 | 279 | 0.254 | 12.5 | 15 | 0.847 | 0.746 | 0.689 |
| **TCGA** | 183 | 4 | 0.022 | 1.9 | 13.9 | 0.966 | 0.966 | -- |
| **NKI** | 152 | 44 | 0.289 | 8.4 | 15 | 0.765 | 0.673 | 0.629 |
| **MAINZ** | 146 | 25 | 0.171 | 7.8 | 15 | 0.892 | 0.802 | 0.706 |
| **VDX** | 119 | 41 | 0.345 | 8.9 | 14.2 | 0.73 | 0.648 | -- |
| **LUND** | 84 | 8 | 0.095 | 7 | 15 | 0.988 | 0.777 | 0.566 |
| **TRANSBIG** | 79 | 18 | 0.228 | 13.7 | 15 | 0.831 | 0.801 | 0.746 |
| **GSE58644** | 52 | 12 | 0.231 | 6.2 | 11.7 | 0.783 | 0.684 | -- |
| **CAL** | 29 | 6 | 0.207 | 8.5 | 12 | 0.816 | 0.762 | -- |
| **DFHCC** | 19 | 2 | 0.105 | 6.1 | 6.8 | 0.895 | -- | -- |
| **UNT** | 19 | 6 | 0.316 | 11.9 | 13.8 | 0.786 | 0.73 | -- |
| **UCSF** | 15 | 1 | 0.067 | 7.3 | 11.6 | 0.917 | 0.917 | -- |

**Supplementary Table 6. Cohort case mix, predicted-risk distribution, and low-risk fractions across training and validation datasets.**

The table summarises cohort-level differences in RSF-defined low-risk fraction, comparator low-risk fractions, predicted-risk distribution, baseline Kaplan–Meier survival, and key clinicopathologic characteristics across *MetaGxBreast*, *GEO*, *SBCC*, and *SCAN-B*. These cohort-level summaries were used to contextualise differences in low-/high-risk proportions across datasets.

DMFS estimates are Kaplan–Meier estimates at the dataset-specific evaluation horizon. The evaluation horizon was 15 years for *MetaGxBreast*, *GEO*, and *SBCC*, and 10 years for *SCAN-B*. Predicted risk refers to RSF-predicted absolute risk at the corresponding dataset-specific horizon. For SCAN-B, no patients remained at risk at the exact 10-year evaluation horizon; therefore, the 10-year baseline DMFS estimate should be interpreted cautiously in relation to follow-up maturity.

| **Cohort** | **n** | **Eval. horizon (years)** | **Baseline DMFS (%)** | **n  at risk** | **Predicted risk, median (IQR)** | **GES  low risk  (%)** | **ACGR  low risk  (%)** | **RSF  low risk  (%)** | **Age  ≥ 65  (%)** | **Tumour  < 2 cm (%)** | **Grade I (%)** | **Node-negative (%)** | **Endocrine therapy  (%)** |
| --- | --- | --- | --- | --- | --- | --- | --- | --- | --- | --- | --- | --- | --- |
| **MetaGxBreast** | 1,997 | 15 | 69.0 | 327 | 0.301  (0.233-0.372) | 11.5 | 33.4 | 33.3 | 42.0 | 41.5 | 18.9 | 69.9 | 52.3 |
| **GEO** | 321 | 15 | 80.4 | 100 | 0.306  (0.246-0.381) | 20.2 | 31.2 | 27.1 | 57.0 | 36.8 | 18.7 | 45.5 | 94.4 |
| **SBCC** | 322 | 15 | 68.3 | 111 | 0.274  (0.212-0.357) | 28.9 | 42.2 | 42.9 | 48.1 | 49.1 | 27.6 | 73.9 | 64.0 |
| **SCAN-B** | 2,492 | 10 | 92.8 | 0 | 0.206  (0.157-0.284) | 32.2 | 44.4 | 68.3 | 71.6 | 70.0 | 26.4 | 76.3 | 86.4 |

**Supplementary Table 7**: **SCAN-B subgroup analysis by age and tumour size**.

Low-risk group sizes and proportions are shown for the standalone gene expression signature, ACGR score, and RSF model within age and tumour-size subgroups. For the RSF model, 10-year DMFS estimates for the low-risk group, high-versus-low risk hazard ratios, C-index, and 10-year AUC are reported. Survival estimates and hazard ratios are shown with 95% confidence intervals. p values correspond to the RSF high-versus-low risk comparison within each subgroup.

| **Subgroup** | **Level** | **n** | **Events (n)** | **Median follow-up (years)** | **GES  low risk**  **n (%)** | **ACGR  low risk**  **n (%)** | **RSF  low risk**  **n (%)** | **RSF low risk 10–year DMFS, % (95% CI)** | **RSF high–vs–low HR (95% CI)** | **p value** |
| --- | --- | --- | --- | --- | --- | --- | --- | --- | --- | --- |
| **Age** | <65 | 707 | 24 | 5.4 | 223 (31.5%) | 444 (62.8%) | 579 (81.9%) | 95.6 (93.0–98.3) | 3.67 (1.64–8.20) | 0.002 |
|  | ≥65 | 1,785 | 84 | 5.2 | 580 (32.5%) | 662 (37.1%) | 1,122 (62.9%) | 96.4 (94.6–98.2) | 5.66 (3.42–9.35) | <0.001 |
| **Tumour size** | <2 cm | 1,745 | 35 | 5.3 | 596 (34.2%) | 963 (55.2%) | 1,405 (80.5%) | 97.5 (96.3–98.8) | 3.95 (2.04–7.66) | <0.001 |
|  | ≥2 cm | 747 | 73 | 5.3 | 207 (27.7%) | 143 (19.1%) | 296 (39.6%) | 90.1 (84.7–95.7) | 2.71 (1.54–4.79) | <0.001 |

| **Subgroup** | **Level** | **n** | **Events (n)** | **Median follow-up (years)** | **C-index GES** | **C-index ACGR** | **C-index RSF** | **10-year AUC GES** | **10-year AUC ACGR** | **10-year AUC RSF** |
| --- | --- | --- | --- | --- | --- | --- | --- | --- | --- | --- |
| **Age** | <65 | 707 | 24 | 5.4 | 0.690 | 0.668 | 0.727 | 0.494 | 0.55 | 0.572 |
|  | ≥65 | 1,785 | 84 | 5.2 | 0.727 | 0.772 | 0.787 | 0.64 | 0.723 | 0.729 |
| **Tumour size** | <2 cm | 1,745 | 35 | 5.3 | 0.704 | 0.680 | 0.722 | 0.585 | 0.614 | 0.638 |
|  | ≥2 cm | 747 | 73 | 5.3 | 0.689 | 0.691 | 0.700 | 0.603 | 0.679 | 0.661 |

**Supplementary Table 8. SCAN-B treatment-stratified performance analysis.**

Performance metrics are shown separately for untreated and endocrine-treated patients in SCAN-B at the 10-year prediction horizon. Discrimination was assessed using C-index and integrated time-dependent AUC (iAUC), prediction error using integrated Brier score (IBS), and risk separation using ΔRMST. Higher C-index, iAUC, and ΔRMST indicate better performance, whereas lower IBS indicates lower prediction error.

| **Subgroup** | **n** | **Events (n)** | **Horizon (years)** | **C-index** | | | **iAUC** | | | **IBS** | | | **ΔRMST** | | |
| --- | --- | --- | --- | --- | --- | --- | --- | --- | --- | --- | --- | --- | --- | --- | --- |
|  |  |  |  | **GES** | **ACGR** | **RSF** | **GES** | **ACGR** | **RSF** | **GES** | **ACGR** | **RSF** | **GES** | **ACGR** | **RSF** |
| **Untreated** | 340 | 11 | 10 | 0.723 | 0.862 | 0.869 | 0.624 | 0.872 | 0.859 | 0.02 | 0.019 | 0.019 | 0.2 | 0.53 | 0.81 |
| **Endocrine therapy** | 2,152 | 97 | 10 | 0.722 | 0.74 | 0.767 | 0.725 | 0.752 | 0.774 | 0.032 | 0.031 | 0.031 | 0.27 | 0.4 | 0.59 |

**Supplementary Table 9. *RSClin*-analogous benchmark in node-negative patients.**

A transparent *RSClin*-analogous Cox comparator was fitted in node-negative *MetaGxBreast* using the published covariate structure of 21-gene RS, age, tumour size, and histological grade, and was applied unchanged to node-negative validation subsets. (A) The table reports C-index point estimates for the standalone 21-gene RS, *RSClin*-analogous comparator, *RSClin*-analogous model plus IgG, and the final RSF model. (B) The table reports the IgG increment using identical IgG-complete patients; in these data, no additional node-negative complete cases were lost after requiring IgG availability. This comparator is not the commercial *RSClin* tool and does not reproduce commercial *RSClin* risk estimates. The Cox models used to estimate the adjusted IgG hazard ratio and perform the likelihood-ratio test retained the prespecified training follow-up; only the reported event counts and C-index estimates were evaluated at 10 years.

**A.** Node-negative C-index benchmark by cohort

| **Cohort** | **Node-negative (n)** | ***RSClin*-complete node-negative  (n)** | **Events ≤10 years (n)** | **21-gene RS  C-index** | ***RSClin*-analogous  C-index** | ***RSClin*-analogous + IgG  C-index** | **ΔC-index  with IgG** | **RSF  C-index** |
| --- | --- | --- | --- | --- | --- | --- | --- | --- |
| **MetaGxBreast** | 1,395 | 1,268 | 242 | 0.639 | 0.659 | 0.676 | +0.017 | 0.718 |
| **GEO** | 146 | 134 | 17 | 0.601 | 0.640 | 0.677 | +0.037 | 0.643 |
| **SBCC** | 238 | 238 | 58 | 0.666 | 0.677 | 0.696 | +0.020 | 0.703 |
| **SCAN-B** | 1,901 | 1,901 | 61 | 0.716 | 0.755 | 0.741 | -0.014 | 0.760 |

**B.** Incremental value of IgG in the MetaGxBreast node-negative training subset

| **Set** | **n** | **Events ≤10 years (n)** | **Adjusted IgG  HR (95% CI)** | **IgG  p value** | ***RSClin*-analogous C-index** | ***RSClin*-analogous + IgG  C-index** | **ΔC-index with IgG** | **LRT χ² (df=1)** | **LRT  p value** |
| --- | --- | --- | --- | --- | --- | --- | --- | --- | --- |
| **MetaGxBreast node-negative IgG-complete** | 1,268 | 242 | 0.64  (0.52–0.78) | <0.0001 | 0.659 | 0.676 | +0.017 | 20.89 | <0.0001 |

# Supplementary Figures

**Supplementary Figure 1**: **Sample-selection workflow across the four dataset groups.**

Flowchart showing the sequential inclusion criteria used to define the final analysis population in each dataset group: (1) tumour samples after duplicate removal; (2) ER-positive/HER2-negative status; (3) no systemic therapy or endocrine therapy only; and (4) availability of required clinicopathologic variables and follow-up data. Colours indicate the four successive selection steps.

**Supplementary Figure 2**: **Gene signature coverage across datasets.**

Heatmap showing the availability of genes included in each evaluated gene expression signature across the training and validation datasets. Orange cells indicate dataset-specific missing genes. The lower panel summarises the percentage of available genes for each signature and lists the missing genes where applicable.

**Supplementary Figure 3**: **Prognostic value of individual candidate features for DMFS.**

Kaplan–Meier curves show distant metastasis-free survival according to individual model features in the training dataset: (**A**) 21-gene RS, (**B**) ROR-S, (**C**) 12-gene MS, (**D**) Amsterdam 70-gene signature, (**E**) 14-gene IgG signature, and (**F**) CCRS. For each feature, patients were dichotomised into low- and high-risk groups using the median value as the cut-off. Low-risk groups are shown in blue and high-risk groups in orange.

| **A**   | **B**   |
| --- | --- |
| **C**   | **D**   |
| **E**   | **F**   |

**Supplementary Figure 4**: **Multivariable Cox associations of gene expression signatures, IgG, and CCRS with DMFS.**

Forest plots show HRs and 95% CIs from multivariable Cox proportional hazards models fitted in the *MetaGxBreast* training dataset. Separate models were fitted for each gene expression signature, 21-gene RS, ROR-S, 12-gene MS, and Amsterdam 70-gene signature, combined with IgG and CCRS. Hazard ratios correspond to standardised continuous predictors.

**Supplementary Figure 5**: **Proportional hazards assessment for multivariable Cox models.**

Scaled Schoenfeld residual plots assess the proportional hazards assumption for Cox models including each established gene expression signature, (A) 21-gene RS, (B) ROR-S, (C) 12-gene MS, and (D) Amsterdam 70-gene signature, together with IgG and CCRS. Points represent scaled Schoenfeld residuals over follow-up time; solid lines show smoothed time-varying coefficient estimates and dashed lines indicate 95% CIs. Global and covariate-specific Schoenfeld test p values are shown for each model.

| **A**   | **B**   |
| --- | --- |
| **C**   | **D**   |

**Supplementary Figure 6**: **Correlations among gene expression signatures, IgG, CCRS, and clinicopathologic variables.**

Heatmaps show pairwise Pearson correlations in the *MetaGxBreast* training dataset. (**A**) Correlations among the evaluated gene expression signatures, IgG, and CCRS. (**B**) Correlations among the evaluated gene expression signatures, IgG, and individual clinicopathologic variables underlying the CCRS. Each cell displays the correlation coefficient, with colour intensity indicating the strength and direction of association.

**A**

**B**

**Supplementary Figure 7**: **Distribution of model feature scores across dataset groups.**

Boxplots with overlaid jittered points show the distributions of the three training-standardised RSF inputs, the cohort-percentile 21-gene RS, IgG signature, and CCRS, across the four datasets (*MetaGxBreast*, *GEO*, *SBCC*, and *SCAN-B*). Each panel corresponds to one feature, and colours denote datasets. Pairwise differences between datasets were evaluated using Wilcoxon rank-sum tests, with p values adjusted using the Benjamini–Hochberg method. Significant adjusted p values are indicated above the corresponding comparisons (p < 0.05, *; p < 0.01, **; p < 0.001, ***; p < 0.0001, ****).

**Supplementary Figure 8**: **Hyperparameter tuning of the clinicogenomic RSF model.**

Out-of-bag (OOB) C-index is shown according to (**A**) the number of trees (*num.trees*) and (**B**) maximum tree depth (*max.depth*) evaluated during RSF model tuning. Dashed red lines indicate the hyperparameter values selected for the final model, which were chosen to maximise OOB C-index in the training dataset.

**A**

**B**

**Supplementary Figure 9**: **Calibration of the 21-gene RS, ACGR score, and RSF model across datasets.**

Calibration plots compare predicted and observed DMFS risk at the selected evaluation horizon: 15 years for *MetaGxBreast*, *GEO*, and *SBCC*, and 10 years for *SCAN-B*. Columns show the 21-gene RS, ACGR score, and RSF model, respectively. The 21-gene RS and ACGR were Cox-recalibrated within each dataset, whereas the RSF model was evaluated using its native predicted risks without recalibration. Points represent grouped predicted and observed risks, and the dashed diagonal line indicates perfect calibration.

| ***MetaGxBreast*** | | |
| --- | --- | --- |
|  |  |  |
| ***GEO*** | | |
|  |  |  |
| ***SBCC*** | | |
|  |  |  |
| ***SCAN-B*** | | |
|  |  |  |

**Supplementary Figure 10**: **Risk stratification by alternative gene expression signatures and RSF models across training and validation datasets.**

Kaplan–Meier curves show DMFS according to low- and high-risk groups in the training and validation datasets. Risk groups are shown for ROR-S (first column), the 12-gene MS (second column), the Amsterdam 70-gene signature (third column), and the RSF model incorporating the cohort-percentile 21-gene RS, IgG signature, and CCRS (fourth column). For each GES, low- and high-risk groups were defined according to the default stratification of the corresponding research-grade implementation. For the RSF model, the training-derived cut-off used in the primary analysis was applied unchanged to all validation datasets. Numbers at risk are shown below each panel.

**Supplementary Figure 11**: **Heterogeneity in DMFS across the original MetaGxBreast training cohorts.**

Kaplan–Meier curves showing distant metastasis-free survival (DMFS) by original cohort within the MetaGxBreast training compendium. The analysis included 1,997 patients from 12 original cohorts contributing to the final training set. The number-at-risk table is shown below the plot at 0, 5, 10, and 15 years. Curves illustrate between-cohort differences in follow-up duration, event burden, and outcome distributions before model development.

**Supplementary Figure 12**: **RSF-defined risk stratification within SCAN-B subgroups.**

Kaplan–Meier curves showing 10-year distant metastasis-free survival according to RSF-defined low- and high-risk groups within SCAN-B subgroups defined by age (<65 vs ≥65 years) (**A**–**B**), and tumour size (<2 vs ≥2 cm) (**C**–**D**). The RSF model was applied without retraining, recalibration, or modification of model parameters, and the same training-derived cut-off used in the primary analysis was applied unchanged. Numbers at risk are shown below each panel. Hazard ratios compare high versus low RSF risk within each subgroup. Ten-year survival estimates should be interpreted in the context of the number of patients remaining at risk near the 10-year horizon.

| **A**   | **B**  |
| --- | --- |
| **C**   | **D**   |

**Supplementary Figure 13**: **RSF-defined risk stratification in SCAN-B according to treatment status.**

Kaplan–Meier curves showing 10-year distant metastasis-free survival according to RSF-defined low- and high-risk groups in untreated (**A**) and endocrine-treated (**B**) SCAN-B patients. The trained RSF model was applied without retraining, recalibration, or modification of model parameters, and the same training-derived cut-off used in the primary analysis was applied unchanged. Numbers at risk are shown below each panel. Hazard ratios compare high versus low RSF risk within each treatment subgroup. Estimates in the untreated subgroup should be interpreted cautiously because of the smaller sample size and limited number of events.

**A**

**B**

**Supplementary Figure 14**: **Time-resolved performance diagnostics of the clinicogenomic RSF model.**

(**A**) Model performance over follow-up time, assessed using the time-dependent Brier score and cumulative/dynamic (C/D) AUC. (**B**) Model diagnostics based on deviance residuals, shown according to event and censoring status.

| **A** |  |
| --- | --- |
| **B** |  |
|  |  |

**Supplementary Figure 15**: **Time-dependent feature importance of the clinicogenomic RSF model.**

(**A**) Permutation-based feature importance over follow-up time, quantified as the loss in cumulative/dynamic AUC after permuting each feature; higher values indicate greater feature importance. (**B**) Aggregated and time-resolved SurvSHAP(t) values summarising overall and time-varying feature contributions to model predictions.

| **A** |  |
| --- | --- |
| **B** |  |

**The REMARK checklist ^5^**

| **Item to be reported** | | **Page no.** |
| --- | --- | --- |
| **INTRODUCTION** | |  |
| 1 | State the marker examined, the study objectives, and any pre-specified hypotheses. | 7 |
| **MATERIALS AND METHODS** | |  |
| *Patients* | |  |
| 2 | Describe the characteristics (e.g., disease stage or co-morbidities) of the study patients, including their source and inclusion and exclusion criteria. | 7-8 |
| 3 | Describe treatments received and how chosen (e.g., randomized or rule-based). | 7-8 |
| *Specimen characteristics* | |  |
| 4 | Describe type of biological material used (including control samples) and methods of preservation and storage. | 7-8 |
| *Assay methods* | |  |
| 5 | Specify the assay method used and provide (or reference) a detailed protocol, including specific reagents or kits used, quality control procedures, reproducibility assessments, quantitation methods, and scoring and reporting protocols. Specify whether and how assays were performed blinded to the study endpoint. | 7-8 |
| *Study design* | |  |
| 6 | State the method of case selection, including whether prospective or retrospective and whether stratification or matching (e.g., by stage of disease or age) was used. Specify the time period from which cases were taken, the end of the follow-up period, and the median follow-up time. | 7-8 |
| 7 | Precisely define all clinical endpoints examined. | 21-22 |
| 8 | List all candidate variables initially examined or considered for inclusion in models. | 10-13 |
| 9 | Give rationale for sample size; if the study was designed to detect a specified effect size, give the target power and effect size. | 21 |
| *Statistical analysis methods* | |  |
| 10 | Specify all statistical methods, including details of any variable selection procedures and other model-building issues, how model assumptions were verified, and how missing data were handled. | 20-23 |
| 11 | Clarify how marker values were handled in the analyses; if relevant, describe methods used for cut point determination. | 20-23 |
| **RESULTS** | |  |
| *Data* | |  |
| 12 | Describe the flow of patients through the study, including the number of patients included in each stage of the analysis (a diagram may be helpful) and reasons for dropout. Specifically, both overall and for each subgroup extensively examined report the numbers of patients and the number of events. | 21 |
| 13 | Report distributions of basic demographic characteristics (at least age and sex), standard (disease-specific) prognostic variables, and tumour marker, including numbers of missing values. | 21 |
| *Analysis and presentation* | |  |
| 14 | Show the relation of the marker to standard prognostic variables. | 22 |
| 15 | Present univariable analyses showing the relation between the marker and outcome, with the estimated effect (e.g., hazard ratio and survival probability). Preferably provide similar analyses for all other variables being analysed. For the effect of a tumour marker on a time-to-event outcome, a Kaplan-Meier plot is recommended. | 22 |
| 16 | For key multivariable analyses, report estimated effects (e.g., hazard ratio) with confidence intervals for the marker and, at least for the final model, all other variables in the model. | 22 |
| 17 | Among reported results, provide estimated effects with confidence intervals from an analysis in which the marker and standard prognostic variables are included, regardless of their statistical significance. | 24-31 |
| 18 | If done, report results of further investigations, such as checking assumptions, sensitivity analyses, and internal validation. | 24-31 |
| **DISCUSSION** | |  |
| 19 | Interpret the results in the context of the pre-specified hypotheses and other relevant studies; include a discussion of limitations of the study. | 32-37 |
| 20 | Discuss implications for future research and clinical value. | 32-37 |

**The STROBE checklist ^6^**

| **Section** | **Item No** | **Recommendation** | **Check** |
| --- | --- | --- | --- |
| **Title and abstract** | 1 | (*a*) Indicate the study’s design with a commonly used term in the title or the abstract | √ |
|  |  | (*b*) Provide in the abstract an informative and balanced summary of what was done and what was found | √ |
| **Introduction** | | | |
| Background / rationale | 2 | Explain the scientific background and rationale for the investigation being reported | √ |
| Objectives | 3 | State specific objectives, including any prespecified hypotheses | √ |
| **Methods** | | | |
| Study design | 4 | Present key elements of study design early in the paper | √ |
| Setting | 5 | Describe the setting, locations, and relevant dates, including periods of recruitment, exposure, follow-up, and data collection | √ |
| Participants | 6 | (*a*) Give the eligibility criteria, and the sources and methods of selection of participants. Describe methods of follow-up | √ |
|  |  | (*b*) For matched studies, give matching criteria and number of exposed and unexposed | -- |
| Variables | 7 | Clearly define all outcomes, exposures, predictors, potential confounders, and effect modifiers. Give diagnostic criteria, if applicable | √ |
| Data sources/ measurement | 8* | For each variable of interest, give sources of data and details of methods of assessment (measurement). Describe comparability of assessment methods if there is more than one group | √ |
| Bias | 9 | Describe any efforts to address potential sources of bias | √ |
| Study size | 10 | Explain how the study size was arrived at | √ |
| Quantitative variables | 11 | Explain how quantitative variables were handled in the analyses. If applicable, describe which groupings were chosen and why | √ |
| Statistical methods | 12 | (*a*) Describe all statistical methods, including those used to control for confounding | √ |
|  |  | (*b*) Describe any methods used to examine subgroups and interactions | √ |
|  |  | (*c*) Explain how missing data were addressed | √ |
|  |  | (*d*) If applicable, explain how loss to follow-up was addressed | √ |
|  |  | (*e*) Describe any sensitivity analyses | √ |
| **Results** | | | |
| Participants | 13* | (a) Report numbers of individuals at each stage of study—e.g. numbers potentially eligible, examined for eligibility, confirmed eligible, included in the study, completing follow-up, and analysed | √ |
|  |  | (b) Give reasons for non-participation at each stage | √ |
|  |  | (c) Consider use of a flow diagram | √ |
| Descriptive data | 14* | (a) Give characteristics of study participants (e.g. demographic, clinical, social) and information on exposures and potential confounders | √ |
|  |  | (b) Indicate number of participants with missing data for each variable of interest | √ |
|  |  | (c) Summarise follow-up time (e.g., average and total amount) | √ |
| Outcome data | 15* | Report numbers of outcome events or summary measures over time | √ |
| Main results | 16 | (*a*) Give unadjusted estimates and, if applicable, confounder-adjusted estimates and their precision (e.g., 95% confidence interval). Make clear which confounders were adjusted for and why they were included | √ |
|  |  | (*b*) Report category boundaries when continuous variables were categorized | √ |
|  |  | (*c*) If relevant, consider translating estimates of relative risk into absolute risk for a meaningful time period | -- |
| Other analyses | 17 | Report other analyses done—e.g. analyses of subgroups and interactions, and sensitivity analyses | √ |
| **Discussion** | | | |
| Key results | 18 | Summarise key results with reference to study objectives | √ |
| Limitations | 19 | Discuss limitations of the study, taking into account sources of potential bias or imprecision. Discuss both direction and magnitude of any potential bias | √ |
| Interpretation | 20 | Give a cautious overall interpretation of results considering objectives, limitations, multiplicity of analyses, results from similar studies, and other relevant evidence | √ |
| Generalisability | 21 | Discuss the generalisability (external validity) of the study results | √ |
| **Other information** | | | |
| Funding | 22 | Give the source of funding and the role of the funders for the present study and, if applicable, for the original study on which the present article is based | √ |
| *Give information separately for exposed and unexposed groups. | | | |

# References

1.	Zhu, H. & Dogan, B.E. American Joint Committee on Cancer's Staging System for Breast Cancer, Eighth Edition: Summary for Clinicians. *Eur J Breast Health* **17**, 234–238 (2021).

2.	Nimeus-Malmstrom, E.*, et al.* Gene expression profiling in primary breast cancer distinguishes patients developing local recurrence after breast-conservation surgery, with or without postoperative radiotherapy. *Breast Cancer Res.* **10**, R34 (2008).

3.	Rueda, O.M.*, et al.* Dynamics of breast-cancer relapse reveal late-recurring ER-positive genomic subgroups. *Nature* **567**, 399–404 (2019).

4.	Thennavan, A.*, et al.* Molecular analysis of TCGA breast cancer histologic types. *Cell Genom* **1**(2021).

5.	McShane, L.M.*, et al.* Reporting recommendations for tumor marker prognostic studies (REMARK). *Journal of the National Cancer Institute* **97**, 1180–1184 (2005).

6.	Vandenbroucke, J.P.*, et al.* Strengthening the Reporting of Observational Studies in Epidemiology (STROBE): explanation and elaboration. *PLoS Med.* **4**, e297 (2007).
